# Proteome-wide analysis of *Coxiella burnetii* for conserved T-cell epitopes with presentation across multiple host species

**DOI:** 10.1186/s12859-021-04181-w

**Published:** 2021-06-02

**Authors:** Lindsay M. W. Piel, Codie J. Durfee, Stephen N. White

**Affiliations:** 1 USDA-ARS Animal Disease Research Unit, Pullman, WA 99164 USA; 2 Department of Veterinary Microbiology and Pathology, Washington State University, Pullman, WA 99164 USA; 3 Center for Reproductive Biology, Washington State University, Pullman, WA 99164 USA

**Keywords:** *Coxiella burnetii*, T-cell epitope, Proteome-wide, Cross-species

## Abstract

**Background:**

*Coxiella burnetii* is the Gram-negative bacterium responsible for Q fever in humans and coxiellosis in domesticated agricultural animals. Previous vaccination efforts with whole cell inactivated bacteria or surface isolated proteins confer protection but can produce a reactogenic immune responses. Thereby a protective vaccine that does not cause aberrant immune reactions is required. The critical role of T-cell immunity in control of *C. burnetii* has been made clear, since either CD8^+^ or CD4^+^ T cells can empower clearance. The purpose of this study was to identify *C. burnetii* proteins bearing epitopes that interact with major histocompatibility complexes (MHC) from multiple host species (human, mouse, and cattle).

**Results:**

Of the annotated 1815 proteins from the Nine Mile Phase I (RSA 493) assembly, 402 proteins were removed from analysis due to a lack of inter-isolate conservation. An additional 391 proteins were eliminated from assessment to avoid potential autoimmune responses due to the presence of host homology. We analyzed the remaining 1022 proteins for their ability to produce peptides that bind MHCI or MHCII. MHCI and MHCII predicted epitopes were filtered and compared between species yielding 777 MHCI epitopes and 453 MHCII epitopes. These epitopes were further examined for presentation by both MHCI and MHCII, and for proteins that contained multiple epitopes. There were 31 epitopes that overlapped positionally between MHCI and MHCII across host species. Of these, there were 9 epitopes represented within proteins containing ≥ 5 total epitopes, where an additional 24 proteins were also epitope dense. In all, 55 proteins were found to contain high scoring T-cell epitopes. Besides the well-studied protein Com1, most identified proteins were novel when compared to previously studied vaccine candidates.

**Conclusion:**

These data represent the first proteome-wide evaluation of *C. burnetii* peptide epitopes. Furthermore, the inclusion of human, mouse, and bovine data capture a range of hosts for this zoonotic pathogen plus an important model organism. This work provides new vaccine targets for future vaccination efforts and enhances opportunities for selecting multiple T-cell epitope types to include within a vaccine.

**Supplementary Information:**

The online version contains supplementary material available at 10.1186/s12859-021-04181-w.

## Introduction

The obligate intracellular bacterium *Coxiella burnetii* is the causative agent of Q fever in humans [1–3]. Centers for Disease Control and Prevention identified this bacterium as a category B agent due to the low infectious dose, environmental stability, and aerosolized spread of the bacterium [2, 4, 5]. Humans infected with *C. burnetii* may present with a variety of different symptoms, ranging from asymptomatic to acute and further to chronic disease [3, 6]. Acute disease is typically characterized by flu-like symptoms, consisting of fever, fatigue, and chills [6]. Individuals which progress to chronic disease most commonly have endocarditis with culture negative blood, where hepatitis and chronic fatigue syndrome have also been described. *C. burnetii* is endemic worldwide, except for New Zealand, and most human outbreaks are blamed on domestic agricultural animals acting as reservoirs of the bacterium [3, 6, 7]. Cows, sheep, and goats represent the main animals of interest, where these animals also contract disease when exposed to *C. burnetii* [1, 5, 6, 8]. Coxiellosis in the small ruminant species, goats and sheep, tends to present with late-term abortions [8, 9]. While cattle may present with late-term abortions, they are more frequently affected by a decrease in calf birthweight or subclinical mastitis [8]. *C. burnetii* is found in large numbers within the placenta of aborted neonates but detection of the bacterium in the urine, milk, uterine fluid, vaginal mucus, and feces of parenteral animals has also occurred [7, 8, 10, 11].

The most widely accepted vaccines against Q fever, or coxiellosis, are known as Q-vax and Coxevac, where the vaccine contains either the Henzerling or Nine Mile Phase I (RSA 493) isolate of *C. burnetii* fixed with formalin [1, 7, 10, 12–14]. These vaccines are not available within the United States [1, 13]. Q-vax is used for human vaccination in Australia and is known to cause adverse side effects in individuals which have had previous exposure to the bacterium [12, 13]. Contrastingly, Coxevac is exploited in Europe for vaccination of agricultural species, wherein this vaccine was used to attempt containment of the 2007–2010 Netherlands outbreak [7, 10]. Either of these vaccination techniques require the producer to culture large amounts of a category B bacterium, a process that is both costly and hazardous [10, 12]. Therefore, investigation into new vaccines has been initiated through isolation of surface antigens or identification of seroreactive proteins [15, 16]. While surface isolated proteins can confer protection, it does not eliminate the cost or safety concerns during product generation.

A clear need exists for low cost, broadly applicable vaccines and especially those that can be produced in safer biosafety level 2 conditions. Subunit vaccines can meet this need, and a new generation of work on *C. burnetii* vaccines has begun based on specific epitope definition. Multiple studies have identified small numbers of epitopes used in human or mouse immune responses, and a few studies have produced subunit vaccines [13, 14, 17–19]. The general conclusion of such work has been that multiple epitopes will be needed to achieve protective immunity [13, 19]. The next challenge is to achieve comprehensive, genome-wide evaluation of potential key epitopes coupled with optimization to achieve broad protection across the multiple host species of this zoonotic pathogen.

Bioinformatic tools have been developed to more quickly and cost effectively assess proteins as host antigens [20–23]. This strategy is known as reverse vaccination development, wherein in silico methods cut down the number of initial screening experiments required to identify putative stimulants of the adaptive immune response [20, 24, 25]. In silico techniques assess the antigenic ability of peptides by modeling their potential immune system interactions as T- or B-cell epitopes [20, 22]. Identification of T-cell epitopes typically evaluates the ability of peptides to be loaded into major histocompatibility complexes, either MHCI or MHCII, wherein both play an important role in the adaptive immune response [21, 22]. MHCI molecules are present on all nucleated host cells and define whether a host cell has been compromised by an invading pathogen [26]. On the other hand, MHCII molecules decorate antigen presenting cells, which function to aid in the initiation of an organized adaptive immune response [21, 22, 27].

Success in the use of T-cell epitope predictors has been seen in rapidly mutating viruses, like HIV and influenza, and in fastidious bacteria [1, 20]. More specifically, the *Brucella mellintensis* protein Omp31 has been of major study during multi-subunit vaccine development against this bacterial agent [28–30]. Research looking into peptide recognition by human monoclonal antibodies isolated similar peptide fragments as B-cell epitope bioinformatic predictors [28, 29]. Additionally, random peptide generation from the Omp31 amino acid sequence allowed for IFN-γ production by T-cells in sheep, wherein the major epitope of interest was bioinformatically determined to be a T-cell epitope in humans later on [29, 30].

For *C. burnetii*, addition of either CD4^+^ or CD8^+^ T lymphocytes alone to infected SCID mice was sufficient to achieve immune control of *C. burnetii* [31]. *C. burnetii* clearance by macrophages has been shown to rely on IFN-γ production by T-cells during the adaptive immune response, which requires accurate loading of antigenic peptides into MHCII molecules for T-cell presentation [13, 15, 21, 32]. Accompanying these data are knockout mouse models that promote the importance of CD8^+^ T-cells in controlling bacterial replication and host tissue pathology, suggesting that MHCI peptide loading also plays an important role during *C. burnetii* infection [27, 31]. Furthermore, it is presumed that cytotoxic T-cells acting on infected host cells degrades availability of the intracellular niche required by this bacterium [27]. While B-cell depletion suggests a role in tissue pathology during *C. burnetii* infection, the inability to link humoral immune responses to restricted bacterial replication suggests that B-cells are not a major player in the control of disease [31, 33]. Thus, this work will focus on identification of T-cell epitopes supporting these beneficial immune responses. Many previous works investigating *C. burnetii* epitopes have focused on known type IV secretion system (T4SS) effectors or proteins eliciting antibody response [14, 17, 19]. The following work will provide the first comprehensive analysis of *C. burnetii* T-cell epitopes on a proteome-wide scale. This will also be one of the few applications to investigate a bacterial proteome, since most prior work has focused on smaller viral proteomes [34]. Furthermore, we will incorporate data from a range of *C. burnetii* isolates to identify conserved epitopes with broad utility and leverage predictions from human, mouse, and ruminant hosts to facilitate development of optimally useful vaccines for this zoonotic pathogen.

## Results

### Conserved *Coxiella burnetii* proteome

*C. burnetii* isolates are genetically diverse, wherein they secrete different type four secretion system effectors, contain antigenic variation, and form a plethora of genomic groups based on multiple loci variable number of tandem repeats analysis (MVLA) [6, 16, 35–37]. For this reason, a proteome-wide comparison between *Coxiella* isolates was completed to ensure pursuit of epitopes within conserved proteins. Nine *Coxiella burnetii* isolates were referenced against Nine Mile Phase I (RSA 493) during proteome-wide comparison. Each strain, with its genomic grouping, tissue of isolation, characteristic of interest, and human virulence, if known, are listed in Table 1. Two genomic group four isolates were chosen based on the observation that this genomic group contains the highest amount of genomic variance between contained isolates [37].

**Table 1:** *C. burnetii* isolates chosen for proteome-wide comparison

Isolate	Genomic Group	Isolation	Special Characteristics	Associated Human Virulence	Reference
Nine Mile Phase I (RSA 493)	I	Tick (United States)	Minimalistic Genome	Acute	[35, 36, 38, 39]
Ohio 314 (RSA 270)	I	Cow Milk (United States)	Cow Isolate Causing Human Disease	Chronic	[36, 39]
Z3055	II-a	Sheep Placenta (Germany)	Netherlands Outbreak of 2007–2010, Non-synonymous Gene Mutations of Membrane Proteins	Acute	[40, 41]
Henzerling	II-b	Human Blood (Italy)	Q-vax Strain	Acute	[36, 39, 40]
701CbB1	III	Cattle (France)	Human PBMC Exposure Causes Similar Cytokine Profile to *C. burnetii* Responsible for Acute Disease	Unknown	[40, 42]
Q545	III	Cattle Abortion (UK)	Type MST20, Common MST in Cow Milk of United States	Unknown	[37, 42, 43]
MSU Goat Q177	IV	Goat Cotyledon (United States)	Putative Ancestral Genotype of Group IV	Chronic	[12, 35, 39, 44]
Schperling	IV	Human Blood		Acute	[37, 40]
CbuG_Q212	V	Heart Valve (Nova Scotia)	Integrated/Chromosomal Plasmid	Chronic	[35, 36, 41]
Dugway 5J108-111	VI	Rodent (United States)	Largest Genome	Avirulent	[35, 39, 41]

Genomic groups were based upon work done by Hemsley et al. [37]. The isolation column states the species and region the *C. burnetii* isolate was derived from. Special characteristics were described when they weighted the decision for study inclusion

**Fig. 1:**
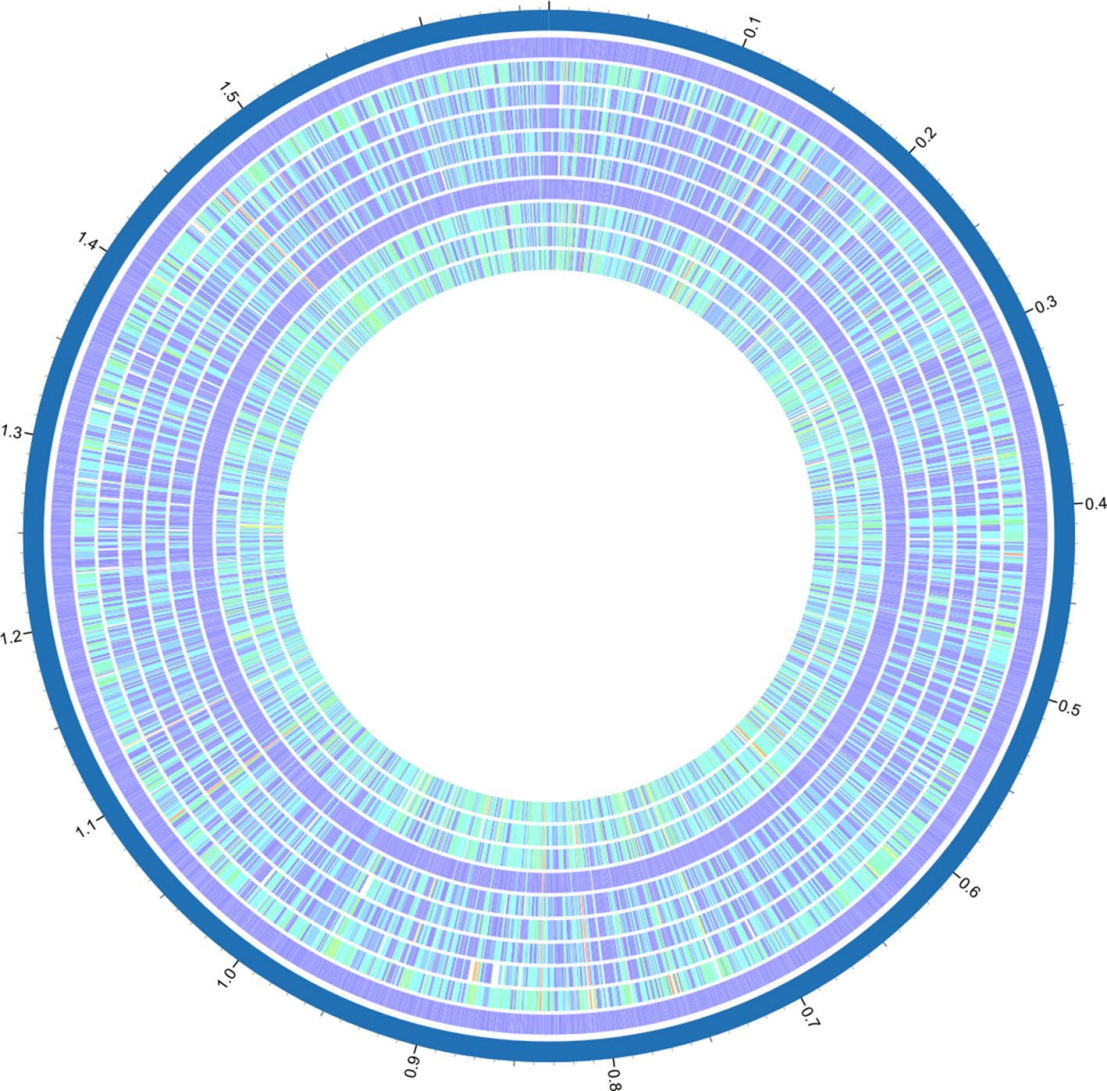
Proteome-wide comparison using Nine Mile Phase I (RSA 493) as a reference strain. The outermost strain is Nine Mile Phase I (RSA 493) and the remainder of the strains moving inward are as follows: CbuG_Q212, Z3055, 701CbB1, Henzerling, Q545, Ohio 314 (RSA 270), Dugway 5J108-111, Schperling, and MSU Goat Q177. The percent identity is indicated by color, where purple-blue is ~ 100–99% identity, green-yellow is ~ 98–70% identity, orange is ~ 69–30% identity, and red is ~ 29–0% identity. Image provided as output from PATRIC database [57]

The tested isolate with the highest percent identity to Nine Mile Phase I (RSA 493) is Ohio 314 (RSA 270) (Fig. 1). This is expected as both isolates belong to genomic group I, indicated by Hemsley et al. [37]. The isolates demonstrating the lowest percent identity compared to Nine Mile Phase I (RSA 493) are Dugway 5J108-111, MSU Goat Q177, Schperling, and CbuG_Q212. The prior strains come from genomic groups IV to VI and represent more divergent isolates as compared to Ohio 314 (RSA 270). Analysis of the overall number of absent or low conservation proteins compared to Nine Mile Phase I (RSA 493) revealed variation between *C. burnetii* isolates (Table 2). In agreement with the pictorial representation of the proteome-wide comparison, less related genomic groups trended towards an increase in the number of absent and unconserved proteins. One exception to this trend was genomic group II-b isolate Z3055, which was missing 201 proteins when compared to Nine Mile Phase I (RSA 493), similar to genomic groups IV-VI. Previous examination of Z3055 has demonstrated that this isolate has an increase in the number of non-synonymous mutations, insertions, and deletions [38, 41].

**Table 2:** Numbers of poorly-conserved proteins between Nine Mile Phase I (RSA 493) and isolates of interest

Isolate	Number of Proteins without a Homolog in Reference Proteome	Number of Proteins Lacking 90% Conservation	Genome Group
Ohio 314 (RSA 270)	55	9	I
Henzerling	81	13	II-a
Z3055	201	19	II-b
701CbB1	76	11	III
Q545	93	16	III
MSU Goat Q177	83	34	IV
Schperling	89	23	IV
CbuG_Q212	215	20	V
Dugway 5J108-111	215	13	VI

Missing or unconserved protein numbers found for isolates compared to Nine Mile Phase I (RSA 493)

A total of 352 proteins were removed upon the basis that the Nine Mile Phase I (RSA 493) proteome lacked a homolog in one of the nine isolates aligned. These predominantly consisted of hypothetical proteins and transposases as opposed to better studied proteins. Overall, proteome-wide comparison between *C. burnetii* isolates and Nine Mile Phase I (RSA 493) resulted in the identification of 1,413 conserved proteins.

### Determination of host homologs in *Coxiella burnetii*

**Fig. 2:**
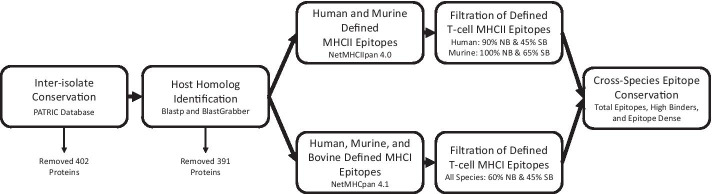
Data Generation and Analysis Flowchart. Steps of data generation are highlighted in larger text. Programs used and data refinement measures are defined in smaller text below

During epitope identification, and future vaccine generation, it is necessary to avoid sensitizing the host’s immune system against itself. Therefore, the resultant protein list was queried using Blastp analysis against the host species of interest (cow, sheep, goat, and human) and the murine disease model for *C. burnetii*. BlastGrabber analysis determined that 391 of 1,413 *C. burnetii* conserved proteins shared homology with species of interest [45]. Thus, the final list of *C. burnetii* proteins for further analysis consisted of 1022 proteins and an overview of the protein selection process can be seen in Fig. 2 (Additional File 1).

### Human and Murine MHCII Epitopes Present in *C. burnetii*

Once a list was generated that contained conserved *C. burnetii* proteins, which lacked host homology, it was possible to exploit NetMHCIIpan 4.0 to define MHCII epitopes. While every murine allele was tested, there were an abundance of human alleles known. To mitigate the number of human alleles, allelic frequency, geographical abundance, and phylogenetic distance were considered (Methods and Additional file 2A/B). In the end, 206 human allelic pairings were chosen to represent common alleles within major clades for MHCII epitope inquiry. Proteome-wide analysis of program derived 15mer peptides returned a total of 293,520 peptides tested. Of these, there were 67,528 peptides that did not bind any of the human alleles. Furthermore, there were 184,615 peptides that did not bind any of the murine alleles. After screening previously identified epitopes to harmonize quality control metrics (Additional files 3 and 4), we found an average binding score of 186 (90%) or strong interaction with 93 (45%) allelic pairings examined during human analysis. On the other hand, the comparison between the datasets for murine analysis delineated an average of 8 (100%) bound alleles or 5 (65%) alleles with strong peptide interaction. Use of these defined numbers to filter the output data returned 1217 and 4072 MHCII epitopes for human and mouse, respectively (Additional file 5). A composite list highlighting MHCII epitopes recognized by both species may be found in Additional file 6 and Fig. 2 summarizes the generation of the composite list. Epitopes that were less than seven amino acids apart were treated as one epitope and the position with the highest human peptide:allele interaction value was retained.

**Table 3:** Human MHCII epitopes with presentation by an exceptional range of host alleles

Pos	GenBank ID	Peptide	NB	WB	SB	Gene Name	Locus Tag	Location
226	AAO89704.2	EGAIRHTHVIPIAGD	206	25	181	*ftsA*	CBU_0140	CYTOPLASM(non-PSE)
259	AAO89704.2	QIKIKYASVLPEEVN	206	28	178	*ftsA*	CBU_0140	CYTOPLASM(non-PSE)
**9**	**AAO90441.1**	**DKEIRAISDYVVNHK**	**206**	**35**	**171**	***prpD***	**CBU_0912**	**CYTOPLASM(non-PSE)**
567	AAO90965.2	QLRIVASHANISGNP	206	74	132		CBU_1468	PSE-Membrane
169	AAO90977.1	RQSIRYYHTAAAIKN	206	10	196		CBU_1480	PSE-Membrane
390	AAO91005.1	RGKFKIYIADPAIAP	206	82	124		CBU_1508	CYTOPLASM(non-PSE)
678	AAO91357.1	GNKIIQIAPARVANR	206	53	153	*parC*	CBU_1866	CYTOPLASM(non-PSE)
403	AAO91392.1	RQNIRAVDTQQVTAA	206	65	141		CBU_1901	SECRETED
329	AAO91494.1	NNAIRYAKNVNVRIQ	206	49	157	*rstB*	CBU_2005	PSE-Membrane
205	AAO91497.1	QGEYIIDIAEALKAK	206	61	145	*argS*	CBU_2008	CYTOPLASM(non-PSE)

Pos indicates the peptide/epitope starting position within the protein sequence. GenBank IDs, gene names, and locus tags are the assembly annotations given on NCBI. NB, WB, and SB represent the total number of alleles bound, the number of alleles bound weakly, and the number of alleles bound strongly by the indicated peptide respectively. Location of the proteins was assigned based on Inmembrane, where PSE designates potentially surface exposed proteins. The bolded row indicates the protein not represented in the murine data when filtering for epitopes binding 100% of alleles tested

Overall, there were 453 peptides, corresponding to 338 total proteins, determined to bind a high number of human and murine alleles or interact with many of the tested alleles strongly. Peptides within this data set that bound to 100% of the tested alleles or proteins that contained greater than or equal to 3 epitopes were isolated to further consolidate the data. Ten peptides bound all 206 human alleles (Table 3). A total of 347 peptides bound all 8 murine alleles (Additional file 7). This is not surprising considering the initial data examination filtered the murine output by focusing on peptides that bound 100% of the alleles analyzed. Marked epitopes within Additional file 7 represent peptides that were one to seven amino acids removed from the epitope observed in Additional file 6; where human peptides with higher binding events were kept during discrepancy in Additional file 6, Additional file 7 retained epitopes that had higher numbers of peptide:allele binding events when considering murine alleles. Of the ten peptides that bound every human allelic pair tested, only one, 9-DKEIRAISDYVVNHK-23 of AAO90441.1 (*prpD*), did not bind all eight murine alleles analyzed.

**Table 4:** MHCII epitope-dense proteins

GenBank ID	Epitope Count	Gene Name	Locus Tag	Location
AAO89616.1	3		CBU_0049	PSE-Membrane
AAO89682.2	3	*ftsI*	CBU_0118	PSE-Membrane
AAO89683.2	3		CBU_0119	PSE-Membrane
AAO89700.1	3	*murC*	CBU_0136	PSE-Membrane
AAO89704.2	3	*ftsA*	CBU_0140	CYTOPLASM(non-PSE)
AAO89832.1	3	*uvrA*	CBU_0274	CYTOPLASM(non-PSE)
AAO89971.1	4		CBU_0419	PSE-Membrane
AAO90011.1	4	*pdhA*	CBU_0461	CYTOPLASM(non-PSE)
AAO90035.1	4		CBU_0486	CYTOPLASM(non-PSE)
AAO90130.1	4		CBU_0586	CYTOPLASM(non-PSE)
AAO90458.2	3	*glpD*	CBU_0931	CYTOPLASM(non-PSE)
AAO90735.2	3		CBU_1226	CYTOPLASM(non-PSE)
AAO90739.1	4		CBU_1230	CYTOPLASM(non-PSE)
AAO90780.1	3		CBU_1273	CYTOPLASM(non-PSE)
AAO90965.2	5		CBU_1468	PSE-Membrane
AAO91193.1	3		CBU_1698	CYTOPLASM(non-PSE)
AAO91233.1	4		CBU_1739	CYTOPLASM(non-PSE)
AAO91303.1	3	*macA*	CBU_1810	PSE-Lipoprotein
AAO91357.1	5	*parC*	CBU_1866	CYTOPLASM(non-PSE)
AAO91378.1	3	*ponA*	CBU_1887	PSE-Membrane

The epitope count designates the number of epitopes present within a protein. NCBI defined information is present in GenBank ID, gene name, and locus tag columns. Location is interpreted from the program Inmembrane, PSE (potentially surface exposed)

Evaluation for epitope dense proteins consisted of data consolidation through isolation of proteins containing a high number of epitopes [24, 46]. Analysis of the 338 proteins with high scoring MHCII-epitopes determined that there were 85 proteins with more than one epitope present. Examination of proteins with three or more epitopes present shortened this list to 20 proteins (Table 4). Notably, three epitope dense proteins also had epitopes that bound every human and murine allele tested; these were AAO89704.2 (*ftsA*), AAO90965.2, and AAO91357.1 (*parC*). Furthermore, AAO90965.2, along with AAO90357.1 (*parC*), encompassed the highest number of epitopes per protein with 5 total epitopes present in either protein.

### Human, murine, and bovine MHCI epitopes

It has become increasingly evident that CD8^+^ T-cells play just as important of a role during resolution of *C. burnetii* infection as CD4^+^ T-cells [27, 31]. While MHCII epitope prediction allows determination of antigenic peptides for CD4^+^ T-cells, there are also MHCI epitope prediction programs available that can help identify antigenic peptides specific for CD8^+^ T-cell recognition [20, 21, 23]. One such program is NetMHCpan 4.1, which has recently been re-trained in its ability to recognize bovine MHCI epitopes, thereby allowing study of another host species of interest [47]. The same list of conserved *C. burnetii* proteins without host-similarity was tested against human, mouse, and bovine MHCI alleles. Similar to NetMHCIIpan 4.0, NetMHCpan 4.1 has a large number of human alleles available for testing. Therefore, phylogenetic trees and geographical frequency of alleles were exploited to alleviate the total number of human alleles run (Methods and Additional file 2C/D), where a total of 82 human alleles were examined during NetMHCpan 4.1 analysis. In addition, we tested all 8 murine alleles and all 105 bovine alleles present on the server.

NetMHCpan 4.1 generates 8-, 9-, 10-, and 11-mer peptides during allele binding assessment, thereby 1,196,564 peptides were generated and tested in their ability to interact with human, murine, and bovine alleles. The number of peptides that did not bind any alleles varied per species and were 783,576; 1,033,923; and 842,516 for human, murine, and bovine respectively. MHCI epitopes have been less widely studied and are therefore less represented in Additional file 4. Accordingly, there were fewer epitopes to aid in the determination as to where the output cut-off values would reside for data filtration. Comparison of these previous epitopes with the present data output determined an average of 51 (62%) bound alleles or a strong interaction with 18 (22%) alleles. While this allowed for a relatively stringent cut-off for the number of peptides binding alleles, the output list was increased by two- to four-fold when peptides that interacted strongly with twenty percent of alleles were included. For this reason, the quantity of alleles strongly bound was restricted to the lower value, 45% of alleles, from MHCII analysis. In examining alleles that bound either 60% of alleles tested or 45% of alleles strongly, there were 1,367 human peptides, 5,355 murine peptides, and 4,438 bovine peptides returned (Additional file 8). As before, the output was searched for duplicate GenBank IDs and positions. A number of returned peptides were only present in murine and bovine analyses, manual annotation thereby allowed for identification of plausible epitopes in all three species tested (Additional file 9).

**Table 5:** Human and bovine MHCI epitopes with presentation by an exceptional range of host alleles

Pos	GenBank ID	Peptide	NB	WB	SB	Gene Name	Locus Tag	Species	Location
213^*^	AAO89864.1	FVAPVTHLF	74	25	49		CBU_0307	Human	SECRETED
54	AAO91456.1	HTFPGVIQL	76	30	46		CBU_1967	Human	MEMBRANE(non-PSE)
113^*^	AAO91555.1	ATYGHIHQM	75	33	42		CBU_2071	Human	MEMBRANE(non-PSE)
33	AAO89719.1	AQSPLLHYL	103	16	87	*pilB*	CBU_0155	Bovine	CYTOPLASM(non-PSE)
132	AAO89740.2	FQIKPPHQL	103	14	89		CBU_0180	Bovine	PSE-Cellwall
101^*^	AAO89868.2	YQYDNVRSV	103	24	79		CBU_0311	Bovine	PSE-Membrane
18	AAO89889.1	AQYPSPQLM	103	14	89	*thiG*	CBU_0333	Bovine	CYTOPLASM(non-PSE)
38	AAO89977.1	SQIENLHKI	103	14	89		CBU_0425	Bovine	CYTOPLASM(non-PSE)
220	AAO90095.2	YQKERVLTF	103	14	89	*rodA*	CBU_0549	Bovine	MEMBRANE(non-PSE)
10	AAO90111.1	TQFEDLPSL	103	21	82		CBU_0567	Bovine	CYTOPLASM(non-PSE)
258	AAO90143.1	AQKEKVFEL	103	15	88	*cysQ-1*	CBU_0599	Bovine	CYTOPLASM(non-PSE)
114	AAO90172.1	RAYEAIQSL	103	15	88	*ppa*	CBU_0628	Bovine	CYTOPLASM(non-PSE)
77	AAO90288.1	FQFTRPHYL	103	11	92		CBU_0748	Bovine	PSE-Lipoprotein
126	AAO90606.1	SQLPVIQKL	103	9	94		CBU_1093	Bovine	MEMBRANE(non-PSE)
91^*^	AAO90780.1	RQYERLIEV	103	20	83		CBU_1273	Bovine	CYTOPLASM(non-PSE)
47	AAO91182.1	AQADRIYEM	103	19	84		CBU_1686	Bovine	CYTOPLASM(non-PSE)
156	AAO91229.2	SQNPALHAL	103	15	88		CBU_1735	Bovine	PSE-Membrane
297	AAO91272.1	SQFDPRKYL	103	15	88	*fbaA*	CBU_1778	Bovine	CYTOPLASM(non-PSE)

NetMHCpan 4.1 defined MHCI epitopes that bound 74–76 (greater than or equal to 90% total) human tested alleles or 103 (98% total) bovine tested alleles. Positions delineated with asterisks indicate that the protein associated is not found within murine data encompassing 98% of bound alleles. Total alleles bound, weak peptide interaction with alleles, and strong peptide interaction with alleles are quantified by NB, WB, and SB respectively. Protein information is outlined in columns containing the GenBank ID, gene name, and locus tag, where this information is defined through Nine Mile Phase I (RSA 493) assembly on NCBI. Pos dictates the peptide’s starting position within the protein of interest and species indicates in which species the peptide was tested for allelic interaction. Location was defined through the use of Inmembrane

**Table 6:** Epitope dense proteins during MHCI epitope analysis

GenBank ID	Epitope Count	Gene Name	Locus Tag	Location
AAO89610.1	4		CBU_0041	CYTOPLASM(non-PSE)
AAO89757.1	6		CBU_0197	PSE-Membrane
AAO89774.2	6		CBU_0215	PSE-Cellwall
AAO89918.2	5		CBU_0364	MEMBRANE(non-PSE)
AAO89941.1	6		CBU_0388	CYTOPLASM(non-PSE)
AAO89977.1	4		CBU_0425	CYTOPLASM(non-PSE)
AAO90093.1	4		CBU_0547	CYTOPLASM(non-PSE)
AAO90229.1	4		CBU_0685	CYTOPLASM(non-PSE)
AAO90293.1	5		CBU_0753	PSE-Membrane
AAO90371.1	4		CBU_0837	PSE-Membrane
AAO90374.1	4	*asnB-2*	CBU_0840	CYTOPLASM(non-PSE)
AAO90423.1	4	*folC*	CBU_0894	CYTOPLASM(non-PSE)
ACI15273.1	6		CBU_1067a	PSE-Membrane
AAO90660.1	4	*mfd*	CBU_1148	CYTOPLASM(non-PSE)
AAO90735.2	4		CBU_1226	CYTOPLASM(non-PSE)
AAO90737.1	4	*qseC*	CBU_1228	PSE-Membrane
AAO90739.1	5		CBU_1230	CYTOPLASM(non-PSE)
AAO90751.1	5		CBU_1242	CYTOPLASM(non-PSE)
AAO90853.1	4	*glmM*	CBU_1350	CYTOPLASM(non-PSE)
AAO90878.1	4	*relA*	CBU_1375	CYTOPLASM(non-PSE)
AAO90965.2	4		CBU_1468	PSE-Membrane
AAO90986.1	4	*lpxH*	CBU_1489	CYTOPLASM(non-PSE)
AAO91142.1	4		CBU_1646	MEMBRANE(non-PSE)
AAO91144.2	4	*dotaA*	CBU_1648	PSE-Membrane
AAO91182.1	7		CBU_1686	CYTOPLASM(non-PSE)
AAO91419.2	4		CBU_1928	PSE-Membrane
AAO91456.1^*^	4		CBU_1967	MEMBRANE(non-PSE)
AAO91467.1	4	*ostA*	CBU_1978	SECRETED

Highly interactive MHCI epitopes that contained greater than or equal to 4 epitopes within all three species studied, human, murine, and bovine. The number of epitopes within a protein is quantified under epitope count. The protein is classified through the GenBank ID, gene name, and locus tag. Inmembrane was exploited to define the location of bacterial proteins. An asterisk next to the GenBank ID indicates that this protein has previously been studied for interaction with the immune system

Data annotation to isolate epitopes represented in human, murine, and bovine species returned 777 MHCI epitopes within 489 different proteins. The data was further evaluated by looking for peptides binding a high number of alleles or for epitope dense proteins. Contrary to MHCII epitope data, there were not any peptides that bound all the bovine or human alleles tested. In order to analyze peptides that bound a high number of alleles tested, the cut-off value was lowered to 98% alleles bound. This returned 17 peptides binding 103 alleles in cattle and 171 peptides binding 8 alleles in the mouse (Table 5 and Additional file 10). This new definition of high allelic binding continued to lack peptide records within the human analysis. The stringency was therefore further lowered to look at peptides that interacted with 90% of the human alleles tested, which led to the identification of 3 human peptides (Table 5). Table 5 shows that highly bound peptides with the most extreme scores do not overlap between the human and bovine species. In comparing human peptides that show exceptional binding to those peptides binding many alleles in the murine species there is only one coinciding protein, AAO91456. Within this shared murine and human protein, the peptide is positionally located at amino acid 54 for human and 261 for the mouse. Contrastingly, the bovine highly bound peptides are predominantly identical to those found within the murine data, where only proteins, AAO89868.2, AAO89977.1, and AAO90780.1, do not coincide. Of these, AAO89868.2 and AAO90780.1 are not represented within the murine data and AAO89977.1 has an epitope present in an alternate position.

**Table 7:** MHCI epitopes that overlapped or are partially contained within MHCII epitopes

Pos	GenBank ID	Peptide	Gene Name	Locus Tag	Location
450	AAO89610.1	SKKT**FSFAKTQALPH**		CBU_0041	CYTOPLASM(non-PSE)
168	AAO89683.2	DSNLKIS**VAHPNNPQ**		CBU_0119	PSE-Membrane
748	AAO89757.1	VRAIRTM**KTSPIVPQ**		CBU_0197	PSE-Membrane
35	AAO89890.1	**AASIITTI**TAQNAEQ	*thiDE*	CBU_0334	CYTOPLASM(non-PSE)
168	AAO89891.1	**PNDYRL**NSAAPYKIS		CBU_0335	CYTOPLASM(non-PSE)
1009	AAO89941.1	K**SIIERVKAL**VSVDK		CBU_0388	CYTOPLASM(non-PSE)
725	AAO90130.1	RSIFVATGAKPNIA**Y**		CBU_0586	CYTOPLASM(non-PSE)
248	AAO90143.1	NPAFVAIGDV**AQKEK**	*cysQ-1*	CBU_0599	CYTOPLASM(non-PSE)
88	AAO90170.1	QGVI**FAAKIGQEL**SR		CBU_0626	CYTOPLASM(non-PSE)
346	AAO90341.1	**ERNITF**ALAVNVSRK		CBU_0807	CYTOPLASM(non-PSE)
316	AAO90458.2	R**SSYAGVRAL**FDDKS	*glpD*	CBU_0931	CYTOPLASM(non-PSE)
194	AAO90577.1	KFN**ILYAHVHRL**AVE		CBU_1063	CYTOPLASM(non-PSE)
209	AAO90684.1	IATYIATTAPRLK**KA**		CBU_1175	MEMBRANE(non-PSE)
48	AAO90696.1	LKAYRWARTHG**AVKK**		CBU_1187	SECRETED
204	AAO90731.2	GKGVISVVAN**VVPKP**	*dapA*	CBU_1222	CYTOPLASM(non-PSE)
254	AAO90737.1	EKR**FTADAAHEL**RTP	*qseC*	CBU_1228	PSE-Membrane
381	AAO90751.1	D**NSFAGVTSL**GVNRP		CBU_1242	CYTOPLASM(non-PSE)
951	AAO90965.2	L**KQWKITHAL**EGGKG		CBU_1468	PSE-Membrane
486	AAO90990.2	KEILYGIET**HPNPSP**		CBU_1493	CYTOPLASM(non-PSE)
226	AAO91047.2	EVSDFEAALA**AARDE**	*ptsP*	CBU_1550	PSE-Membrane
59	AAO91155.2	HKKWRAI**SSHPVAIV**		CBU_1659	MEMBRANE(non-PSE)
31	AAO91229.2	AVEIRGINVATVA**VS**		CBU_1735	PSE-Membrane
82	AAO91240.2	VRAIKAI**YAFENGRG**	*sspB*	CBU_1746	CYTOPLASM(non-PSE)
152	AAO91245.1	TNRRNIQSLIA**EADP**		CBU_1751	CYTOPLASM(non-PSE)
33	AAO91303.1	KDRQIIVA**KLQPSVT**	*macA*	CBU_1810	PSE-Lipoprotein
138	AAO91320.2	NKVLRHVSVA**FQEDP**		CBU_1827	CYTOPLASM(non-PSE)
118	AAO91322.1	RQH**YTASTPEQL**MQQ	*lolB*	CBU_1829	PSE-Lipoprotein
122	AAO91396.2	DDVINTL**SSNPLPPV**	*ftsX*	CBU_1905	PSE-Membrane
140^*^	AAO91401.1	Q**SQYAAKVSL**AAAKQ	*com1*	CBU_1910	SECRETED
121	AAO91474.1	DPDF**YTIKPIVSM**RS		CBU_1985	PSE-Membrane
191	AAO91493.1	LSERYAAVVNQLK**KQ**		CBU_2004	CYTOPLASM(non-PSE)

The entire peptide defined within the column delineates the MHCII-epitope while the MHCI-epitope is represented by bolded and underlined areas within the MHCII epitope. Pos indicates the starting position within the protein amino acid sequence. The row with an asterisk next to the positional number indicates that the protein was encountered during review of previous *C. burnetii* research

In studying MHCI epitopes for epitope dense proteins, we found a higher number of epitopes per protein (7 in AAO91182.1) was achieved as compared to a maximum of 5 MHCII epitopes (Table 6). There were 28 proteins classified as epitope dense when assessing the MHCI epitope data for proteins with four or more epitopes. Of the epitope dense proteins identified, there was one present in the human analysis, twenty-one present in mouse data, and two present in bovine analysis when comparing the proteins identified as containing epitopes with high allelic coverage (Table 5 and Additional file 10). Human analysis identified CBU_1967, where cattle analysis contained proteins CBU_0425 and CBU_1686. The epitope dense proteins that were missing in the murine high allelic output were CBU_0685, CBU_1226, CBU_1228 (*qseC*), CBU_1242, CBU_1489 (*lpxH*), CBU_1928, and CBU_1978 (*ostA*).

### Consolidation of epitopes or proteins from MHCI and MHCII data

Assessment of the *C. burnetii* proteome for both MHCI and MHCII epitopes enables identification of multi-use epitopes and proteins. There were 31 epitopes that had overlapping use by MHCI and MHCII (Table 7). Of these epitopes, only one has been previously studied and is present in Additional file 4; this is Com1 (CBU_1910) [9, 13, 14, 17–19]. Other notable aspects were that some of the epitopes constituted a complete overlap whereas others were mildly overlapped. In total, eleven of the thirty-one epitopes completely overlapped between identified MHCI and MHCII epitopes. Furthermore, Inmembrane predicted that approximately fifty percent of the epitopes were cytoplasmic and that the remaining fifty percent were in some way associated with the bacterial membrane.

GenBank IDs from MHCI and MHCII output summary tables, Additional files 6 and 9, were combined to determine if additional epitope dense proteins would be observed. The resultant proteins can be seen in Table 8, where 33 epitope dense proteins were identified with at least 5 epitopes. Seven of these proteins were not previously identified when looking at either MHCI or MHCII epitope dense proteins alone (GenBankIDs are AAO89890.1 (*thiDE*), AAO90155.1 (*yaeT*), AAO90323.2, AAO90990.2, AAO91128.1 (*icmO*), AAO91393.1, and AAO91455.1 (*hemA*)). Additionally, there were 19 proteins absent from the combined epitopes dense protein list that were previously encompassed in either the MHCI or MHCII data. Many of the proteins which were lost in the combined epitope dense protein table represent proteins containing the number of epitopes near the bottom of the previous cut-off values. None of the previously studied proteins in Additional file 4 were present as an epitope dense protein in the unified MHCI and MHCII Table 8. Nine of the epitope dense proteins also contained overlapping epitopes; however, these epitopes were considered separate during quantification due to their binding alternate immune major histocompatibility complexes. In comparing MHCI and MHCII epitope results it was possible to elucidate epitopes or proteins that could stimulate both cytotoxic T-cells and T-helper cells.

**Table 8:** Proteins with ≥ 5 epitopes present overall for MHCI and MHCII

GenBank ID	Pos	Peptide	Gene Name	Locus Tag	T-cell Epitope	Location
AAO89610.1	72	YLKKHLESL		CBU_0041	MHCI	CYTOPLASM(non-PSE)
AAO89610.1	450	SKKT**FSFAKTQALPH**		CBU_0041	MHCII	CYTOPLASM(non-PSE)
AAO89610.1	453	**FSFAKTQAL**		CBU_0041	MHCI	CYTOPLASM(non-PSE)
AAO89610.1	459	**QALPH**LWEL		CBU_0041	MHCI	CYTOPLASM(non-PSE)
AAO89610.1	523	KVADTHIAF		CBU_0041	MHCI	CYTOPLASM(non-PSE)
AAO89757.1	95	HADNIKIVL		CBU_0197	MHCI	PSE-Membrane
AAO89757.1	146	NQIEFNHAL		CBU_0197	MHCI	PSE-Membrane
AAO89757.1	164	QPYPVNVYL		CBU_0197	MHCI	PSE-Membrane
AAO89757.1	374	HVHTPVHRL		CBU_0197	MHCI	PSE-Membrane
AAO89757.1	636	RAILKPTTF		CBU_0197	MHCI	PSE-Membrane
AAO89757.1	748	VRAIRTM**KTSPIVPQ**		CBU_0197	MHCII	PSE-Membrane
AAO89757.1	754	**KTSPIVPQ**L		CBU_0197	MHCI	PSE-Membrane
AAO89774.2	37	RIYRPLFSL		CBU_0215	MHCI	PSE-Cellwall
AAO89774.2	59	AADDSTISL		CBU_0215	MHCI	PSE-Cellwall
AAO89774.2	283	LAAIIHTKTNVIDDQ		CBU_0215	MHCII	PSE-Cellwall
AAO89774.2	354	HMATVITTL		CBU_0215	MHCI	PSE-Cellwall
AAO89774.2	420	YLIEKGHHL		CBU_0215	MHCI	PSE-Cellwall
AAO89774.2	487	LQYPEDPSL		CBU_0215	MHCI	PSE-Cellwall
AAO89774.2	508	YLDELPNYL		CBU_0215	MHCI	PSE-Cellwall
AAO89890.1	33	H**AASIITTI**	*thiDE*	CBU_0334	MHCI	CYTOPLASM(non-PSE)
AAO89890.1	35	**AASIITTI**TAQNAEQ	*thiDE*	CBU_0334	MHCII	CYTOPLASM(non-PSE)
AAO89890.1	70	TLPPTVIKL	*thiDE*	CBU_0334	MHCI	CYTOPLASM(non-PSE)
AAO89890.1	215	SAISSAIAL	*thiDE*	CBU_0334	MHCI	CYTOPLASM(non-PSE)
AAO89890.1	384	HTLYELSRAHAIQPS	*thiDE*	CBU_0334	MHCII	CYTOPLASM(non-PSE)
AAO89918.2	6	VQNPTLESL		CBU_0364	MHCI	MEMBRANE(non-PSE)
AAO89918.2	173	MAFHLPHAL		CBU_0364	MHCI	MEMBRANE(non-PSE)
AAO89918.2	233	AVATPVQKL		CBU_0364	MHCI	MEMBRANE(non-PSE)
AAO89918.2	276	KAISPHASL		CBU_0364	MHCI	MEMBRANE(non-PSE)
AAO89918.2	281	HASLLKHTL		CBU_0364	MHCI	MEMBRANE(non-PSE)
AAO89941.1	421	DSYPIIQSL		CBU_0388	MHCI	CYTOPLASM(non-PSE)
AAO89941.1	488	MAFEILEQL		CBU_0388	MHCI	CYTOPLASM(non-PSE)
AAO89941.1	1009	**SIIERVKAL**		CBU_0388	MHCI	CYTOPLASM(non-PSE)
AAO89941.1	1009	K**SIIERVKAL**VSVDK		CBU_0388	MHCII	CYTOPLASM(non-PSE)
AAO89941.1	1248	VAAPLFMTL		CBU_0388	MHCI	CYTOPLASM(non-PSE)
AAO89941.1	1264	RMFAKV**FSL**		CBU_0388	MHCI	CYTOPLASM(non-PSE)
AAO89941.1	1270	**FSL**PIEVEL		CBU_0388	MHCI	CYTOPLASM(non-PSE)
AAO89977.1	9	VVDS**KPHEL**		CBU_0425	MHCI	CYTOPLASM(non-PSE)
AAO89977.1	13	**KPHEL**TLLF		CBU_0425	MHCI	CYTOPLASM(non-PSE)
AAO89977.1	37	SQIENLHKIL		CBU_0425	MHCI	CYTOPLASM(non-PSE)
AAO89977.1	84	NATDFEYSETQPIET		CBU_0425	MHCII	CYTOPLASM(non-PSE)
AAO89977.1	140	SQLFRTIDAILVKTS		CBU_0425	MHCII	CYTOPLASM(non-PSE)
AAO89977.1	197	TVYDTTITL		CBU_0425	MHCI	CYTOPLASM(non-PSE)
AAO90011.1	58	HTPYLNTIPAETEAQ	*pdhA*	CBU_0461	MHCII	CYTOPLASM(non-PSE)
AAO90011.1	171	SSYPHPFLM	*pdhA*	CBU_0461	MHCI	CYTOPLASM(non-PSE)
AAO90011.1	362	DPQKVYAAYTEAMKD	*pdhA*	CBU_0461	MHCII	CYTOPLASM(non-PSE)
AAO90011.1	508	IRDRIVPIVADEART	*pdhA*	CBU_0461	MHCII	CYTOPLASM(non-PSE)
AAO90011.1	636	HQDSHNLLM	*pdhA*	CBU_0461	MHCI	CYTOPLASM(non-PSE)
AAO90011.1	843	AKMVVYTALKALADQ	*pdhA*	CBU_0461	MHCII	CYTOPLASM(non-PSE)
AAO90093.1	90	IAYDQAIQL		CBU_0547	MHCI	CYTOPLASM(non-PSE)
AAO90093.1	124	KAYQKAIAL		CBU_0547	MHCI	CYTOPLASM(non-PSE)
AAO90093.1	382	LQYQVPQKL		CBU_0547	MHCI	CYTOPLASM(non-PSE)
AAO90093.1	521	SKKYIIALIKRNNFK		CBU_0547	MHCII	CYTOPLASM(non-PSE)
AAO90093.1	549	KAIEGYLVL		CBU_0547	MHCI	CYTOPLASM(non-PSE)
AAO90130.1	99	FAFKKFYVL		CBU_0586	MHCI	CYTOPLASM(non-PSE)
AAO90130.1	574	VQAYYIAQVEKTARR		CBU_0586	MHCII	CYTOPLASM(non-PSE)
AAO90130.1	725	RSIFVATGAKPNIA**Y**		CBU_0586	MHCII	CYTOPLASM(non-PSE)
AAO90130.1	738	**Y**VYEHKGTF		CBU_0586	MHCI	CYTOPLASM(non-PSE)
AAO90130.1	799	HPVFHGSVVKAIASA		CBU_0586	MHCII	CYTOPLASM(non-PSE)
AAO90130.1	967	MAAAHLRSL		CBU_0586	MHCI	CYTOPLASM(non-PSE)
AAO90130.1	984	GHRVFYVALIEKAEE		CBU_0586	MHCII	CYTOPLASM(non-PSE)
AAO90155.1	257	DKKHVYITIHLVEGP	*yaeT*	CBU_0611	MHCII	PSE-Membrane
AAO90155.1	320	GDRGYAFARVNVIPT	*yaeT*	CBU_0611	MHCII	PSE-Membrane
AAO90155.1	469	SQYQQNYSF	*yaeT*	CBU_0611	MHCI	PSE-Membrane
AAO90155.1	550	IAAPSVLAF	*yaeT*	CBU_0611	MHCI	PSE-Membrane
AAO90155.1	794	FQFSFGVSL	*yaeT*	CBU_0611	MHCI	PSE-Membrane
AAO90229.1	497	PVDIKYDTNNLAQSA		CBU_0685	MHCII	CYTOPLASM(non-PSE)
AAO90229.1	738	VVSPVPPVL		CBU_0685	MHCI	CYTOPLASM(non-PSE)
AAO90229.1	784	YAKPILHPM		CBU_0685	MHCI	CYTOPLASM(non-PSE)
AAO90229.1	855	SAYPATDRLYF		CBU_0685	MHCI	CYTOPLASM(non-PSE)
AAO90229.1	940	TAAEVQWRL		CBU_0685	MHCI	CYTOPLASM(non-PSE)
AAO90293.1	97	YGVNVYEVANQIRDK		CBU_0753	MHCII	PSE-Membrane
AAO90293.1	265	SADQSIVTL		CBU_0753	MHCI	PSE-Membrane
AAO90293.1	453	QAANIFRSF		CBU_0753	MHCI	PSE-Membrane
AAO90293.1	602	KAFDSVFAM		CBU_0753	MHCI	PSE-Membrane
AAO90293.1	785	VVLPHYNHL		CBU_0753	MHCI	PSE-Membrane
AAO90293.1	999	YAYKFKLFL		CBU_0753	MHCI	PSE-Membrane
AAO90323.2	57	AANDFAIKL		CBU_0789	MHCI	CYTOPLASM(non-PSE)
AAO90323.2	278	QLEIQRQKAEAANKA		CBU_0789	MHCII	CYTOPLASM(non-PSE)
AAO90323.2	481	TIFEHFSRL		CBU_0789	MHCI	CYTOPLASM(non-PSE)
AAO90323.2	694	EQFIFRAKAEKEAKS		CBU_0789	MHCII	CYTOPLASM(non-PSE)
AAO90323.2	816	KAIEAFLKM		CBU_0789	MHCI	CYTOPLASM(non-PSE)
ACI15273.1	10	YSSEIPQNL		CBU_1067a	MHCI	PSE-Membrane
ACI15273.1	63	IAAPLPIQL		CBU_1067a	MHCI	PSE-Membrane
ACI15273.1	105	RQFQPLATL		CBU_1067a	MHCI	PSE-Membrane
ACI15273.1	193	AQFTDPITF		CBU_1067a	MHCI	PSE-Membrane
ACI15273.1	280	YLKEIVTVL		CBU_1067a	MHCI	PSE-Membrane
ACI15273.1	535	FMRDGVLSL		CBU_1067a	MHCI	PSE-Membrane
AAO90660.1	70	SADTPILHF	*mfd*	CBU_1148	MHCI	CYTOPLASM(non-PSE)
AAO90660.1	212	VEKIESVRLLPAREY	*mfd*	CBU_1148	MHCII	CYTOPLASM(non-PSE)
AAO90660.1	531	KIYVPVSSL	*mfd*	CBU_1148	MHCI	CYTOPLASM(non-PSE)
AAO90660.1	665	VAVLVPTTL	*mfd*	CBU_1148	MHCI	CYTOPLASM(non-PSE)
AAO90660.1	768	TATPIPRTL	*mfd*	CBU_1148	MHCI	CYTOPLASM(non-PSE)
AAO90735.2	42	FIRLYYAHVALEDIK		CBU_1226	MHCII	CYTOPLASM(non-PSE)
AAO90735.2	81	EVKIRVFNPQLDRDG		CBU_1226	MHCII	CYTOPLASM(non-PSE)
AAO90735.2	160	STLEAPISM		CBU_1226	MHCI	CYTOPLASM(non-PSE)
AAO90735.2	339	FIGLYTSDVYRSDPR		CBU_1226	MHCII	CYTOPLASM(non-PSE)
AAO90735.2	921	AADKGTATF		CBU_1226	MHCI	CYTOPLASM(non-PSE)
AAO90735.2	1015	AAFDHRHIF		CBU_1226	MHCI	CYTOPLASM(non-PSE)
AAO90735.2	1484	GTAPLFHAL		CBU_1226	MHCI	CYTOPLASM(non-PSE)
AAO90737.1	149	TLYDPATEL	*qseC*	CBU_1228	MHCI	PSE-Membrane
AAO90737.1	254	EKR**FTADAAHEL**RTP	*qseC*	CBU_1228	MHCII	PSE-Membrane
AAO90737.1	256	**FTADAAHEL**	*qseC*	CBU_1228	MHCI	PSE-Membrane
AAO90737.1	418	RVFERFFRM	*qseC*	CBU_1228	MHCI	PSE-Membrane
AAO90737.1	467	VTFPLIHNF	*qseC*	CBU_1228	MHCI	PSE-Membrane
AAO90739.1	8	QALDPQQSF		CBU_1230	MHCI	CYTOPLASM(non-PSE)
AAO90739.1	312	RMKDLLSQL		CBU_1230	MHCI	CYTOPLASM(non-PSE)
AAO90739.1	411	FQDTSIIQF		CBU_1230	MHCI	CYTOPLASM(non-PSE)
AAO90739.1	568	QLIEITPAL		CBU_1230	MHCI	CYTOPLASM(non-PSE)
AAO90739.1	581	DIPFHAVEIEKLAHR		CBU_1230	MHCII	CYTOPLASM(non-PSE)
AAO90739.1	745	AKNPIQIMTIHKAKG		CBU_1230	MHCII	CYTOPLASM(non-PSE)
AAO90739.1	806	KADPVYNYL		CBU_1230	MHCI	CYTOPLASM(non-PSE)
AAO90739.1	827	ITRLLYVAATRAKES		CBU_1230	MHCII	CYTOPLASM(non-PSE)
AAO90739.1	1038	RWIIDYKSATPNDEP		CBU_1230	MHCII	CYTOPLASM(non-PSE)
AAO90751.1	22	FAFINPAEL		CBU_1242	MHCI	CYTOPLASM(non-PSE)
AAO90751.1	55	VTIPTGLSF		CBU_1242	MHCI	CYTOPLASM(non-PSE)
AAO90751.1	129	RVNDISPE**F**		CBU_1242	MHCI	CYTOPLASM(non-PSE)
AAO90751.1	137	**F**AFSFSPKF		CBU_1242	MHCI	CYTOPLASM(non-PSE)
AAO90751.1	381	**NSFAGVTSL**		CBU_1242	MHCI	CYTOPLASM(non-PSE)
AAO90751.1	381	D**NSFAGVTSL**GVNRP		CBU_1242	MHCII	CYTOPLASM(non-PSE)
AAO90780.1	90	RQYERLIEVF		CBU_1273	MHCI	CYTOPLASM(non-PSE)
AAO90780.1	100	KAHDIGYVF		CBU_1273	MHCI	CYTOPLASM(non-PSE)
AAO90780.1	159	VAKYIAVSTQEAALD		CBU_1273	MHCII	CYTOPLASM(non-PSE)
AAO90780.1	287	NFKYHWAVADYLQRA		CBU_1273	MHCII	CYTOPLASM(non-PSE)
AAO90780.1	399	VPDYVTLKNQLVAKK		CBU_1273	MHCII	CYTOPLASM(non-PSE)
AAO90853.1	65	SAAGVNIKL	*glmM*	CBU_1350	MHCI	CYTOPLASM(non-PSE)
AAO90853.1	133	DKPMKTVVADRLGKA	*glmM*	CBU_1350	MHCII	CYTOPLASM(non-PSE)
AAO90853.1	162	STFPSNLTL	*glmM*	CBU_1350	MHCI	CYTOPLASM(non-PSE)
AAO90853.1	187	VAPSIFHEL	*glmM*	CBU_1350	MHCI	CYTOPLASM(non-PSE)
AAO90853.1	364	VMVKHPQVL	*glmM*	CBU_1350	MHCI	CYTOPLASM(non-PSE)
AAO90878.1	140	KLSERLTTL	*relA*	CBU_1375	MHCI	CYTOPLASM(non-PSE)
AAO90878.1	193	YLNPNEYSL	*relA*	CBU_1375	MHCI	CYTOPLASM(non-PSE)
AAO90878.1	280	TALSIVHAL	*relA*	CBU_1375	MHCI	CYTOPLASM(non-PSE)
AAO90878.1	306	DNGYRSIHTAVIGPE	*relA*	CBU_1375	MHCII	CYTOPLASM(non-PSE)
AAO90878.1	428	KMVPLTRTL	*relA*	CBU_1375	MHCI	CYTOPLASM(non-PSE)
AAO90965.2	108	RLFPGHVWL		CBU_1468	MHCI	PSE-Membrane
AAO90965.2	168	NIDIYYHTAEGQLIP		CBU_1468	MHCII	PSE-Membrane
AAO90965.2	235	SQWESSYFL		CBU_1468	MHCI	PSE-Membrane
AAO90965.2	366	YQPKRIQTLF		CBU_1468	MHCI	PSE-Membrane
AAO90965.2	567	QLRIVASHANISGNP		CBU_1468	MHCII	PSE-Membrane
AAO90965.2	608	ANGFKFLKAAPLSVA		CBU_1468	MHCII	PSE-Membrane
AAO90965.2	700	PIAFHIATLNPSSQS		CBU_1468	MHCII	PSE-Membrane
AAO90965.2	951	**KQWKITHAL**		CBU_1468	MHCI	PSE-Membrane
AAO90965.2	951	L**KQWKITHAL**EGGKG		CBU_1468	MHCII	PSE-Membrane
AAO90990.2	255	KPYEPILNL		CBU_1493	MHCI	CYTOPLASM(non-PSE)
AAO90990.2	369	MASPHVASL		CBU_1493	MHCI	CYTOPLASM(non-PSE)
AAO90990.2	426	YPPRIVANTVAFNAK		CBU_1493	MHCII	CYTOPLASM(non-PSE)
AAO90990.2	486	KEILYGIET**HPNPSP**		CBU_1493	MHCII	CYTOPLASM(non-PSE)
AAO90990.2	494	**HPNPSP**TIF		CBU_1493	MHCI	CYTOPLASM(non-PSE)
AAO91128.1	103	RQAQGIYYF	*icmO*	CBU_1632	MHCI	PSE-Membrane
AAO91128.1	174	KVFSIVRSM	*icmO*	CBU_1632	MHCI	PSE-Membrane
AAO91128.1	447	YAVEGFAVVPAQARS	*icmO*	CBU_1632	MHCII	PSE-Membrane
AAO91128.1	571	VRGKFFYADPKRTKH	*icmO*	CBU_1632	MHCII	PSE-Membrane
AAO91128.1	734	QAMNIAVEL	*icmO*	CBU_1632	MHCI	PSE-Membrane
AAO91182.1	46	AQAD**RIYEM**		CBU_1686	MHCI	CYTOPLASM(non-PSE)
AAO91182.1	50	**RIYEM**LQQL		CBU_1686	MHCI	CYTOPLASM(non-PSE)
AAO91182.1	201	KQIPLITRY		CBU_1686	MHCI	CYTOPLASM(non-PSE)
AAO91182.1	224	SILDVFLQL		CBU_1686	MHCI	CYTOPLASM(non-PSE)
AAO91182.1	265	RLIDNRFSF		CBU_1686	MHCI	CYTOPLASM(non-PSE)
AAO91182.1	512	SQQEKTIQL		CBU_1686	MHCI	CYTOPLASM(non-PSE)
AAO91182.1	618	SVNEHANQF		CBU_1686	MHCI	CYTOPLASM(non-PSE)
AAO91357.1	259	GEIVITALPHQVSGN	*parC*	CBU_1866	MHCII	CYTOPLASM(non-PSE)
AAO91357.1	376	FVIERLHLL	*parC*	CBU_1866	MHCI	CYTOPLASM(non-PSE)
AAO91357.1	439	EKKIRDEQAILTKER	*parC*	CBU_1866	MHCII	CYTOPLASM(non-PSE)
AAO91357.1	516	KGWIRAAKGHEVEGE	*parC*	CBU_1866	MHCII	CYTOPLASM(non-PSE)
AAO91357.1	678	GNKIIQIAPARVANR	*parC*	CBU_1866	MHCII	CYTOPLASM(non-PSE)
AAO91357.1	739	PRGFRKVDNVAVDEN	*parC*	CBU_1866	MHCII	CYTOPLASM(non-PSE)
AAO91378.1	79	PKPLIHAVLATEDAR	*ponA*	CBU_1887	MHCII	PSE-Membrane
AAO91378.1	103	ISIIRAAKAVILTGK	*ponA*	CBU_1887	MHCII	PSE-Membrane
AAO91378.1	253	TAKYHAATTQVKAPY	*ponA*	CBU_1887	MHCII	PSE-Membrane
AAO91378.1	539	YAIEYLTRF	*ponA*	CBU_1887	MHCI	PSE-Membrane
AAO91378.1	552	NVLPHSLSL	*ponA*	CBU_1887	MHCI	PSE-Membrane
AAO91393.1	104	TADDFTVYF		CBU_1902	MHCI	SECRETED
AAO91393.1	116	SADQLPVAF		CBU_1902	MHCI	SECRETED
AAO91393.1	286	YALDVLSTL		CBU_1902	MHCI	SECRETED
AAO91393.1	365	EEELKRVKAQVIAQN		CBU_1902	MHCII	SECRETED
AAO91393.1	410	VKNIEAVTAQQIQQV		CBU_1902	MHCII	SECRETED
AAO91419.2	10	QVISLTHQF		CBU_1928	MHCI	PSE-Membrane
AAO91419.2	297	SAYGKTLNM		CBU_1928	MHCI	PSE-Membrane
AAO91419.2	504	IQFKGPSAM		CBU_1928	MHCI	PSE-Membrane
AAO91419.2	594	TEKLIIVAETKEDKK		CBU_1928	MHCII	PSE-Membrane
AAO91419.2	667	SAFWQTIKL		CBU_1928	MHCI	PSE-Membrane
AAO91455.1	145	TKRIRSETAIGA**NPV**	*hemA*	CBU_1966	MHCII	CYTOPLASM(non-PSE)
AAO91455.1	157	**NPV**SIAYAVVQLAKR	*hemA*	CBU_1966	MHCII	CYTOPLASM(non-PSE)
AAO91455.1	227	RLSDIPTYL	*hemA*	CBU_1966	MHCI	CYTOPLASM(non-PSE)
AAO91455.1	383	ILHQPTTKL	*hemA*	CBU_1966	MHCI	CYTOPLASM(non-PSE)
AAO91455.1	394	AAYEDQVQL	*hemA*	CBU_1966	MHCI	CYTOPLASM(non-PSE)
AAO91467.1	163	VADKGTL**TL**	*ostA*	CBU_1978	MHCI	SECRETED
AAO91467.1	170	**TL**YPKTAIL	*ostA*	CBU_1978	MHCI	SECRETED
AAO91467.1	615	FSFEQLFAL	*ostA*	CBU_1978	MHCI	SECRETED
AAO91467.1	730	KADIRYLFVHGNEDS	*ostA*	CBU_1978	MHCII	SECRETED
AAO91467.1	832	TAYGFELQL	*ostA*	CBU_1978	MHCI	SECRETED

The epitope type is defined in the T-cell epitope column. Protein information is outlined in the following columns: GenBank ID, gene name, and locus tag, where this information is defined through Nine Mile Phase I (RSA 493) assembly on NCBI. Pos dictates the peptides starting position within the protein of interest and location was defined through the use of Inmembrane

## Discussion

We sought to leverage both *C. burnetii* and host genomic diversity to predict widely useful T-cell epitopes across a range of hosts for this zoonotic pathogen. Epitopes were identified by leveraging an array of MHCII and MHCI alleles for antigen presentation, thereby capturing epitopes incorporated in both MHC systems across multiple host species. The results highlight broadly useful epitopes, including many with minimal prior study, that can be used for future work and vaccine development.

Foundational data aimed to capture broad representation of *C. burnetii* and focus on proteins that would avoid self-reactive antigens. In particular, we selected at least one sequence from each genomic group (Table 1), including the relatively minimal genome of virulent Nine Mile Phase I (RSA 493) as a reference. This resulted in a refined list of 1413 conserved proteins for further analysis. This list was further screened for homology within human, mouse, and ruminant host proteins to avoid stimulating potential autoimmune responses. 391 such proteins were identified, suggesting large-scale use of host protein domain structures by *C. burnetii*. During assembly of the protein query list, it became apparent that a substantial number of annotated genes within the Nine Mile Phase I (RSA 493) genome lack discovery work and that many underlying functions are suggested by homology to alternate bacterial proteins. This promotes analyzing the bacterial proteome in its entirety, as the importance of many *C. burnetii* proteins has yet to be determined.

Relatively few Gram-negative bacteria have been examined for T-cell epitopes on a proteome-wide basis [34], leaving much of the previous epitope studies examining effector proteins or proteins residing at the cellular surface [24, 48–50]. This is no exception for studies examining *C. burnetii* proteins for host cell epitopes, wherein previous work has focused on proteins injected into the host cytoplasm by the type four secretion system (T4SS) or proteins which elicit an antibody response [13, 14, 17]. Resolution of *C. burnetii* infection is known to rely on the production of a Th1 type immune response that results in the production of IFN-γ [15, 32, 33]. This immune response is accomplished by coordination of T-helper cells through interaction with MHC class II peptide loaded molecules and a harmonized cytokine environment [22]. Therefore proteome-wide analysis for *C. burnetii* contained epitopes began with identifying MHC class II interacting peptides (See Repository). The MHC class II analysis herein identified numerous epitopes with relatively high allelic interactions (Additional file 6), many with cross-species presentation (Additional file 7). Some had presentation by an exceptional range of host alleles (Table 3), and many were clustered in epitope dense proteins of special interest (Table 4). Studies looking at the importance of different immune cellular subsets during *C. burnetii* infection has led to increased interest in CD8^+^ T-cell stimulation, which requires MHC class I presentation of peptides [27, 31]. As such, similar methodology was implemented to identify epitopes binding an exceptional number of host MHC class I alleles (Table 5 and Additional file 8) and epitope dense proteins characterized by MHC class I binding (Table 6).

The Dugway 5J108-111 isolate of *C. burnetii* represents the only known avirulent strain included in the following analysis and was included to exemplify the high degree of genomic variability contained between bacterial isolates [37, 39, 41]. Discarding the Dugway 5J108-111 isolate would result in the addition of thirteen proteins to the analysis, where two would be removed upon identification of host homologs (Additional file 12A). Examination of the remaining eleven proteins determined that their inclusion would minimally alter the data included herein, as only three new MHCI T-cell epitopes with cross-species representation were discerned (Additional file 12B). Notably, none of these additional epitopes bound an exceptional number of alleles tested nor did they encompass epitope dense proteins.

Examination of either the MHC class I or II datasets demonstrates the return of proteins which have not previously been studied for T-cell epitopes. As mentioned before, much of the earlier work identifying T-cell epitopes has focused on certain protein subsets [9, 13, 14, 16, 19]. Therefore, return of novel epitope-containing proteins does not preclude epitopes defined within this work; instead, these epitopes may represent more immunogenic peptides that exemplify a range of host species. For example, a group of novel epitope-containing proteins can be seen within the MHC class II and I datasets and are responsible for bacterial cell division, encompassing AAO89704.2 (*ftsA*), AAO89682.2 (*ftsI*), and AAO90095.2 (*rodA*) [51]. The MHC class I analysis for bacterial epitopes supports the addition of a ruminant species to the dataset. It is believed that many human outbreaks arise from domestic ruminants, consisting of sheep, goats, and cattle, therefore vaccination efforts in ruminants may help in the prevention of zoonotic spread [3, 6, 7]. Furthermore, coxiellosis in animals does not come without consequence, where sheep and goats present most frequently with late-term abortions and cattle have decreased birthing weights and possible mastitis [8]. Consequently, *Coxiella burnetii* infection in these species causes clear economic losses and requires intervention.

A potential pitfall of bioinformatic analysis of T-cell epitopes is the possibility of false positives [14, 21, 52]. This hinderance has been largely combated through the inclusion of more MHC ligand elution data during server training [21, 23, 47]. During this research, alleviation of false positives was attempted by assessing a plethora of different MHCI and MHCII alleles and investigating the peptides which had high allelic coverage. It is presumed that false positives arise due to a lack of training data between alleles and that analysis of a myriad of alleles would promote dilution of false positives [21, 47, 52]. When considering the 8 murine alleles tested during use of either NetMHCpan 4.1 or NetMHCIIpan 4.0, as compared to either 82-206 human alleles or 105 bovine alleles, it is noticeable that there were an increasing number of peptides falling within the filtered data sets (Additional files 6 and 8). This data is suspected to contain a number of false positives, but comparison with high binding peptides of human and cattle alleles is believed to lessen this burden. Previous research on *C. burnetii* defined T-cell epitopes have used methodologies that measure the ability to achieve host T-cell activation in response to epitopes of interest; including EliSpot, ELISA, flow cytometry, and peptide loading into MHCs [13, 14, 18, 19]. It remains imperative to test returned T-cell epitopes for their ability to interact with the host immune system before production of vaccine candidates may begin.

Once data had been acquired for both MHC class I and II alleles, it became possible to cross-analyze outputs. Investigation into overlapping MHC class II and I epitopes defined 31 peptides of interest (Table 7). Com1, a well-studied *C. burnetii* protein of interest, was represented within this output. Importantly, former analysis of Com1 as a vaccine candidate against *C. burnetii* has demonstrated a decent amount of promise [13, 18, 19]. Specifically, mice exposed to Com1 were afforded better protection during challenge assays and produced IFN-γ during immune system stimulation. Unfortunately, Com1 was categorized as a secreted protein by Inmembrane, where it is a well-studied surface associated protein [16, 18, 36]. It is likely that there is a secondary processing step that is not recognized by Inmembrane. This does not disqualify the overall purpose for such notation, as many vaccination efforts have focused on surface proteins, where it is believed that these proteins most readily interact with the immune system during infection [1, 25, 53]. While care should be taken regarding protein location, proteins residing at the level of the membrane or that are secreted would suggest improved immune recognition.

Com1 did not remain in the MHC class I and II cross-analysis when assessing for epitope dense proteins (Table 8). Likewise, none of the previously studied proteins present in Additional file 4 are represented in the 33 epitope dense proteins composed from MHC class I and II data. Of these novel epitope-containing proteins, there were seven that were not returned when assessing MHC class I or II epitope dense proteins alone. These are AAO89890.1 (*thiDE*), AAO90155.1 (*yaeT*), AAO90323.2, AAO90990.2, AAO91128.1 (*icmO*), AAO91393.1, and AAO91455.1 (*hemA*), which represent epitope rich proteins that have a balanced MHC class I and II coverage. Three of the previously mentioned proteins are designated as secreted or membrane exposed proteins by Inmembrane, AAO90155.1 (*yaeT*), AAO91128.1 (*icmO*), and AAO91393.1. Therefore, these proteins are suggested to more readily interact with the immune system upon arrival of the bacterium within host tissues. IcmO and YaeT are significant proteins in regards to host:pathogen interaction as IcmO is part of the multi-subunit T4SS and YaeT is responsible for assembly of beta-barrel surface proteins [54–56].

Cross-analysis between MHC class I and II data allows for future vaccination efforts to cover both classes of T-cell epitopes. Furthermore, the investigation herein also aids in epitope decision with regards to alternate vaccine types. For instance, identified epitope dense proteins provide a source of epitopes which can partake in a vectored vaccine [20, 34]. On the other hand, when looking at proteins that contain overlapping MHCI and MHCII epitopes, there is the possibility of using the epitopes in a heterologous recombinant subunit vaccine. As a result, the provided data allows for vaccination efforts against *Coxiella burnetii* to move forward without restrictions on the approach to be used.

## Conclusions

These data represent the first comprehensive, proteome-wide examination of T-cell epitopes for *C. burnetii*. The use of multiple divergent *C. burnetii* isolates enabled the identification of widely conserved proteins and epitopes to empower future work. Furthermore, the use of multiple host species for antigen presentation analyses supports the existence of widely conserved epitopes that can be broadly useful across many host species for this zoonotic pathogen. The specific results highlight many proteins and epitopes not previously described in regards to host immune recognition, and in so doing provide useful direction for future work in developing epitope-rich vaccines.

## Methods

### Proteome-wide comparison between *Coxiella burnetii* isolates

The PATRIC database (Pathosystems Resource Integration Center) was exploited to run proteome-wide comparisons between *C. burnetii* isolates (https://www.patricbrc.org/) [57, 58]. Bacterial isolates selected and their corresponding assembly numbers are as follows: Nine Mile Phase I (RSA 493) (ASM776v2), Dugway 5J108-111 (ASM1710v1), MSU Goat Q177 Priscilla (ASM16887v3), CbuG_Q212 (ASM1986v1), Z3055 (Z3055), 701CbB1 (ASM263396v1), Henzerling (ASM263402v1), Schperling (ASM263406), Q545 (ASM289675v1), and Ohio 314 (RSA 270) (ASM224728v1) [37, 38, 59–62]. Of these, Nine Mile Phase I (RSA 493), MSU Goat Q177, and Schperling updated assemblies were not loaded into the PATRIC database. These three proteomes were downloaded from the National Center for Biotechnology Information (NCBI) database as multi-FASTA files. Nine Mile Phase I (RSA 493) was chosen as the reference strain during analysis because of its short genome length and well-documented virulence [38, 39]. An E-value of 1e^−8^ was used, where proteins were considered homologs if the percent identity was 90% or above [37, 63].

### Homolog identification in the host species

Nine Mile Phase I (RSA 493) proteins found to be conserved between *C. burnetii* isolates were entered as a multi-FASTA file onto the Blastp server and analyzed for homologs present in host species. The host species tested and their taxonomic Id’s are as follows human (txid 9606), mouse (txid 10,088), cow (txid 9913), goat (txid 9925), and sheep (txid 9940). BlastGrabber was exploited to analyze results obtained from NCBI’s basic local alignment search tool (BLASTp) [45]. An E-value cut-off of 0.01 (1e^−2^) and a percent identity greater than 35% was set based on previous experimental methods used to remove host homologs from analysis [24, 63, 64].

### Phylogenetic analysis for human MHC alleles

The top ten most common MHCI alleles for eleven global regions were determined using the Allele Frequency Net Database (AFND) (http://www.allelefrequencies.net/default.asp) [65, 66]. Duplicate alleles were removed from the resultant list and protein FASTA sequences were obtained from the International Immunogenetics Information System/Human Leukocyte Antigen (IMGT/HLA) database (https://www.ebi.ac.uk/ipd/imgt/hla/) [67]. Of the remaining MHCI alleles, there were three allelic FASTA sequences that were no longer available within the database and were therefore excluded going forward; these were A*29:25, A*29:50, and A*02:264. Phylogenetic trees were built using MEGA X, wherein 1,000 bootstraps were run during the construction of both a neighbor-joining and maximum likelihood tree [68]. Afterwards, the trees were condensed so that only bootstrap values above 80 were involved in branch generation (Additional file 2C/D). If MHCI alleles were closely related, then a representative allele was chosen based upon its representation within the annotated geographic regions denoted by the AFND. There were 83 human MHCI alleles chosen for epitope analysis from NetMHCpan 4.1. The MHCII DRB1 locus has annotated data for the top ten alleles for each of the eleven geographic regions on AFND. Contrastingly, the DPA1, DPB1, DQA1, and DQB1 loci did not have region associated data. Alleles in these alternate loci were chosen based on an allelic frequency that was greater than or equal 0.05 in any one geographic region, where the database was filtered for gold and silver data that were obtained from available literature [65]. Protein FASTA sequences were again obtained from the IMGT/HLA database. Notably, DRB1*04:140, DRB1*04:155, DRB1*12:09, DPB1*26:01:01, DPB1*101:01, DQA1*05:02, and DQB1 02:03:01 MHCII alleles were partial sequences and were removed from further analysis. MEGA X was used to make a neighbor-joining and maximum likelihood tree with the remaining MHCII alleles using a minimum of 999 bootstraps per analysis (Additional file 2A/B) [68]. The remainder of the MHCII analysis was completed as described above for the MHCI analysis. There were 28 DRB1, 4 DPA1, 27 DPB1, 10 DQA1, and 7 DQB1 alleles chosen for epitope inquiry, governing a total of 206 allelic parings.

### Identification of human, murine, and bovine MHC epitopes

Conserved Nine Mile Phase I (RSA 493) proteins lacking homology to host species were loaded onto the NetMHCpan 4.1 database for analysis across multiple host species (https://services.healthtech.dtu.dk/service.php?NetMHCpan-4.1) and (http://www.cbs.dtu.dk/services/NetMHCpan/) [23, 47, 69] Of the approximately 3,000 human MHCI alleles, 83 were chosen based upon locus frequency within defined populations, representation of alleles in more than one region, and greater evolutionary distance as discerned by phylogenetic tree analysis. During this investigation it was determined that allele B*13:07 N was not available for assessment on NetMHCpan 4.1, decreasing the number of human alleles assessed to 82. There were 8 murine MHCI alleles present, which sought to represent the available inbred strains of lab mice. Lastly, 105 BoLA (bovine leukocyte antigens) MHCI alleles were recently trained for server inclusion and allowed for representation of a host ruminant species. Each of these MHCI allelic groupings were evaluated over the course of multiple program runs. A complete list of tested MHCI alleles can be found in Additional file 11. The threshold values were set at 0.5 for %Rank of a strong binder and 2 for %Rank of a weak binder during the assessment. Peptide length was kept at the baseline parameters, wherein this gave 8-, 9-, 10-, and 11-mer peptides in the output.

NetMHCIIpan 4.0 was exploited to study peptides that can bind human or murine MHCII alleles (https://services.healthtech.dtu.dk/service.php?NetMHCIIpan-4.0) [21, 23, 70]. There were 8 murine MHCII alleles and 936 human MHCII alleles present on the given server, which generates thousands of human MHCII complexes. Human MHCII alleles to be tested were chosen based on the previously mentioned phylogenetic analysis. Threshold values identified a strong binder as a %Rank less than 2.0 and a weak binder as a %Rank greater than or equal to 2.0 and less than or equal to 10.0. The standard peptide length of 15 amino acids was kept during this investigation. A complete list of tested MHCII alleles can be found in Additional file 11. Positional output differed by one amino acid base between NetMHCIIpan 4.0 and NetMHCpan 4.1 (starting positions designated as 0 versus 1); therefore, all output data was standardized to achieve consistent positional designation.

### *C. burnetii* proteome localization

The multi-FASTA file that contained conserved bacterial and nonhomologous host proteins was run through Inmembrane to determine each protein’s localization within the bacterium [71]. The program coordinates runs for a combination of bioinformatic tools consisting of TMHMM, SignalP, LipoP, and HMMER [72–75].

## Supplementary Information

**Additional file 1**. ***C. burnetii*** **proteins lacking host homologs and containing inter-isolate conservation**. A FASTA format list of the* C. burnetii* proteins studied for T-cell epitopes.

**Additional file 2**. **Allelic phylogenetic analysis**. Phylogenetic trees containing MHCII (A and B) or MHCI (C and D) alleles from human species. MHCII alleles were included based on geographical representation for the DRB1 locus or an allelic frequency of 0.05 or greater for the remaining loci. MHCI alleles were included based on geographical representation as denoted by AFND. 999 bootstraps were run during neighbor-joining tree generation for MHCII alleles (A), while 1,000 bootstraps were completed when producing the maximum likelihood tree for MHCII alleles (B). MHCI allelic comparison using either the neighbor-joining (C) or the maximum likelihood method (D) using 1,000 bootstraps. Trees were condensed to only show branching when bootstrap values were 80 or above.

**Additional file 3**.** Isolation of quality controlled MHCII and MHCI epitopes**. Contains the methodology used to select output epitopes of interest.

**Additional file 4**.** Previously studied** ***Coxiella burnetii* epitopes**. Locus tag and gene name based upon genomic assembly annotations for Nine Mile Phase I (RSA 493) on National Center for Biotechnology and Information (NCBI). Species indicates which host or model organism the epitope was analyzed for. The epitope type column describes if the peptide studied was in regards to a B-cell or T-cell (MHCI or MHCII) epitope. If more than one epitope was isolated, then the epitope types are separated by backslashes to indicate the order of MHC epitopes or an ampersand to indicate B-cell production of antibodies. Locus tag superscripts denote protein subcellular location and if the protein was disqualified from NetMHCpan due to previous analysis. 1 is for membrane associated, 2 is for cytoplasmic location, 3 is for unknown location, and an asterisk indicates removal. Epitope amino acid positions are annotated to represent the pre-processed forms of the proteins

**Additional file 5**.** MHCII epitopes with high scoring allelic interactions**. MHCII epitopes found to bind either 186 (90% total) human alleles or 8 (100% total) murine alleles. Data also includes MHCII epitopes found to interact strongly with 90 (45% total) human alleles or 5 (65% total) murine alleles. NCBI retrieved information includes the GenBank ID, gene name, and locus tag. Location of given proteins was determined through use of the program Inmembrane, where PSE represents a potential surface exposed protein. The peptide column contains the 15mer peptides generated by NetMHCIIpan 4.0 for MHCII binding assessment, position of peptide start within a protein is indicated within the pos (position) column. NB, WB, and SB represent peptide and MHCII allele interactions, where NB is the total number of alleles bound, WB signifies weak binders, and SB represents strong binders. Species dictates the animal in which the alleles tested originated from.

**Additional file 6**.** Condensed MHCII epitopes**. Pos indicates the position within the protein in which the NetMHCIIpan 4.0 15mer generated peptide begins. GenBank ID, gene name, and locus tag are protein specific information originating from NCBI. Location of proteins was annotated using the Inmembrane program, where PSE stands for potentially surface exposed protein.

**Additional file 7**.** Murine MHCII epitopes with exceptional allelic coverage**. Rows with asterisks present next to the position number indicate a peptide shift from epitopes defined in Additional Table 4, where peptides bound more murine alleles when the 15mer was shifted one to two amino acids over. Pos indicates the starting position of the peptide of interest. Protein identification is determined by NCBI annotated information given by the GenBank ID, gene name, or locus tag. NB, WB, and SB described the character of peptide:MHCII allele interaction, where total alleles bound, weak binding, and strong binding are respectively defined. Inmembrane was used to define protein location within the bacterium

**Additional file 8**.** MHCI epitopes with high allelic interactions**. NetMHCpan 4.1 designated MHCI epitopes within human, murine, and bovine species, wherein epitopes bound 60% of alleles tested or interacted strongly with 45% of alleles tested for each species. Position of peptide start is defined in the pos column. GenBank ID, gene name, and locus tag were defined by Nine Mile Phase I (RSA 493) assembly on NCBI. NB, WB, and SB represent number of alleles bound, weak binders, and strong binders respectively. The species defines what animal the alleles were being tested for and location was designated by Inmembrane.

**Additional file 9**.** Condensed MHCI epitopes**. Manually annotated high binding MHCI epitopes that are present in all three species, human, murine, and bovine. Pos indicates the peptide of interest start site within the NCBI cited protein (GenBank ID, gene name, or locus tag). Location was derived through use of the program Inmembrane, wherein PSE defines a potentially surface exposed protein.

**Additional file 10**.** Murine MHCI epitopes with exceptional allelic binding**. MHCI epitopes that bound 8 (98% of total) alleles in the murine species. Rows with positions labeled by asterisks and bolded text represent epitopes that are different in position as compared to human MHCI epitopes that bind 74 to 76 alleles; while rows with asterisks and underlined text represent peptides that vary in position between murine and bovine epitopes that bind 98% of tested alleles. Pos describes the starting position of the peptide within the protein. Identification of the protein is given through the NCBI obtained GenBank ID, gene name, and locus tag. Location was defined by the Inmembrane program, where PSE is an acronym for potentially surface exposed. The number of alleles bound by a peptide are indicated in the NB column. If the peptide:MHCI interaction was weak it was quantified as WB and if the peptide:MHCI interaction was strong it was quantified as SB.

**Additional file 11**.** MHCI and MHCII tested alleles**. (**A**) MHCI alleles tested during the use of NetMHCpan 4.1. Human, murine, and bovine alleles are notated HLA, H-2, and BoLA respectively. (**B**) MHCII alleles tested during exploitation of NetMHCIIpan 4.0. Murine and human alleles are designated by H-2 or HLA respectively. Notably, the human DRA1 locus is not highly variable, therefore only the DRB1 allele for this pairing changed. Otherwise, each of the DPA1 and DQA1 loci were paired and tested with each of their respective DPB1 and DQB1 loci.

**Additional file 12**.** Exclusion of Dugway 5J108-111**. (**A**) Protein GenBank IDs returned to analysis when Dugway 5J108-111 was removed from inter-isolate comparison. Homology to host species is noted in the second column, where a yes indicates removal of the protein before T-cell epitope analysis. (**B**)* C. burnetii* defined MHCI T-cell epitopes represented within human, murine, and bovine species during Dugway 5J108-111 exclusion. Pos indicates the position at which the peptide begins within the protein of interest. GenBank ID, gene name, and locus tag provide protein identification parameters present in assembly ASM776v2. Protein localization was defined through the use of Inmembrane. Program updates labeled the location of AAO91013.1 as IM+peri (inner membrane plus the periplasmic space), this was altered to Membrane (non-PSE) to keep with location labels in the remainder of the manuscript.

## Data Availability

The datasets generated and/or analysed during the current study are available in the Open Science Framework repository at https://osf.io/rn6qa/ with accession RN6QA.

## Abbreviations

MHC: Major histocompatibility complex
BoLA: Bovine leukocyte antigens
PATRIC: Pathosystems Resource Integration Center
NCBI: National Center for Biotechnology and Information
BLAST: Basic local alignment search tool
AFND: Allele Frequency Net Database
IMGT/HLA: International Immunogenetics Information System/Human Leukocyte Antigen
MVLA: Multiple loci variable number of tandem repeats analysis
PSE: Potentially surface exposed
T4SS: Type four secretion system

## References

[1] Beare PA, Chen C, Bouman T, Pablo J, Unal B, Cockrell DC (2008). Candidate antigens for Q fever serodiagnosis revealed by immunoscreening of a Coxiella burnetii protein microarray. Clin Vaccine Immunol.

[2] Heinzen RA, Hackstadt T, Samuel JE (1999). Developmental biology of Coxiella burnettii. Trends Microbiol.

[3] Raoult D, Marrie T, Mege J (2005). Natural history and pathophysiology of Q fever. Lancet Infect Dis.

[4] Tigertt WD, Benenson AS, Gochenour WS (1961). Airborne Q fever. Bacteriol Rev.

[5] Welsh HH, Lennette EH, Abinanti FR, Winn JF (1958). Air-borne transmission of Q fever: the role of parturition in the generation of infective aerosols. Ann N Y Acad Sci.

[6] Maurin M, Raoult D (1999). Q fever. Clin Microbiol Rev.

[7] Hogerwerf L, van den Brom R, Roest HI, Bouma A, Vellema P, Pieterse M (2011). Reduction of Coxiella burnetii prevalence by vaccination of goats and sheep. The Netherlands Emerg Infect Dis.

[8] Agerholm JS (2013). Coxiella burnetii associated reproductive disorders in domestic animals: a critical review. Acta Vet Scand.

[9] Vigil A, Ortega R, Nakajima-Sasaki R, Pablo J, Molina DM, Chao CC (2010). Genome-wide profiling of humoral immune response to Coxiella burnetii infection by protein microarray. Proteomics.

[10] Arricau-Bouvery N, Souriau A, Bodier C, Dufour P, Rousset E, Rodolakis A (2005). Effect of vaccination with phase I and phase II Coxiella burnetii vaccines in pregnant goats. Vaccine.

[11] Rodolakis A, Berri M, Hechard C, Caudron C, Souriau A, Bodier CC (2007). Comparison of Coxiella burnetii shedding in milk of dairy bovine, caprine, and ovine herds. J Dairy Sci.

[12] Marmion BP, Ormsbee RA, Kyrkou M, Wright J, Worswick DA, Izzo AA (1990). Vaccine prophylaxis of abattoir-associated Q fever: eight years' experience in Australian abattoirs. Epidemiol Infect.

[13] Chen C, Dow C, Wang P, Sidney J, Read A, Harmsen A, et al. Identification of CD4+ T cell epitopes in C. burnetii antigens targeted by antibody responses. PLoS One. 2011;6(3):e17712. 10.1371/journal.pone.0017712 PMC3057979 21423609

[14] Scholzen A, Richard G, Moise L, Baeten LA, Reeves PM, Martin WD (2019). Promiscuous Coxiella burnetii CD4 Epitope Clusters Associated With Human Recall Responses Are Candidates for a Novel T-Cell Targeted Multi-Epitope Q Fever Vaccine. Front Immunol.

[15] Shannon JG, Heinzen RA (2009). Adaptive immunity to the obligate intracellular pathogen Coxiella burnetii. Immunol Res.

[16] Zhang G, Samuel JE (2004). Vaccines against Coxiella infection. Expert Rev Vaccines.

[17] Jaydari A, Forouharmehr A, Nazifi N (2019). Determination of immunodominant scaffolds of Com1 and OmpH antigens of Coxiella burnetii. Microb Pathog.

[18] Xiong X, Meng Y, Wang X, Qi Y, Li J, Duan C (2012). Mice immunized with bone marrow-derived dendritic cells stimulated with recombinant Coxiella burnetii Com1 and Mip demonstrate enhanced bacterial clearance in association with a Th1 immune response. Vaccine.

[19] Xiong X, Qi Y, Jiao J, Gong W, Duan C, Wen B. Exploratory study on Th1 epitope-induced protective immunity against Coxiella burnetii infection. PLoS One. 2014;9(1):e87206. 10.1371/journal.pone.0087206 PMC3907486 24498044

[20] Soria-Guerra RE, Nieto-Gomez R, Govea-Alonso DO, Rosales-Mendoza S (2015). An overview of bioinformatics tools for epitope prediction: implications on vaccine development. J Biomed Inform.

[21] Reynisson B, Barra C, Kaabinejadian S, Hildebrand WH, Peters B, Nielsen M (2020). Improved prediction of MHC II antigen presentation through integration and motif deconvolution of mass spectrometry MHC eluted ligand data. J Proteome Res.

[22] Sanchez-Trincado JL, Gomez-Perosanz M, Reche PA (2017). Fundamentals and methods for T- and B-cell epitope prediction. J Immunol Res.

[23] Reynisson B, Alvarez B, Paul S, Peters B, Nielsen M. NetMHCpan-4.1 and NetMHCIIpan-4.0: improved predictions of MHC antigen presentation by concurrent motif deconvolution and integration of MS MHC eluted ligand data. Nucleic Acids Res. 2020;48(W1):W449-W54. 10.1093/nar/gkaa379 PMC7319546 32406916

[24] Hisham Y, Ashhab Y. Identification of Cross-Protective Potential Antigens against Pathogenic Brucella spp. through Combining Pan-Genome Analysis with Reverse Vaccinology. J Immunol Res. 2018;2018:1474517. 10.1155/2018/1474517 PMC6304850 30622973

[25] Fiuza TS, Lima J, de Souza GA (2020). EpitoCore: mining conserved epitope vaccine candidates in the core proteome of multiple bacteria strains. Front Immunol.

[26] Turvey SE, Broide DH (2010). Innate immunity. J Allergy Clin Immunol.

[27] Buttrum L, Ledbetter L, Cherla R, Zhang Y, Mitchell WJ, Zhang G. Both Major Histocompatibility Complex Class I (MHC-I) and MHC-II Molecules Are Required, while MHC-I Appears To Play a Critical Role in Host Defense against Primary Coxiella burnetii Infection. Infect Immun. 2018;86(4). 10.1128/IAI.00602-17 PMC5865044 29311245

[28] Li J, Hu F, Chen S, Luo P, He Z, Wang W (2017). Characterization of novel Omp31 antigenic epitopes of Brucella melitensis by monoclonal antibodies. BMC Microbiol.

[29] Pan Pang FZ, Bin J, Ming L, Jinwei H, Ping J, Rongjiong Z, Jianbing D, Yuexin Z. Bioinformatics analysis of T- and B-combined epitopes of OMP31 protein of Brucella melitensis in Xinjiang, China. Int J Clin Exp Med. 2017;10(9):13320–30.

[30] Wang W, Wu J, Qiao J, Weng Y, Zhang H, Liao Q (2014). Evaluation of humoral and cellular immune responses to BP26 and OMP31 epitopes in the attenuated Brucella melitensis vaccinated sheep. Vaccine.

[31] Read AJ, Erickson S, Harmsen AG (2010). Role of CD4+ and CD8+ T cells in clearance of primary pulmonary infection with Coxiella burnetii. Infect Immun.

[32] Dellacasagrande J, Capo C, Raoult D, Mege JL (1999). IFN-gamma-mediated control of Coxiella burnetii survival in monocytes: the role of cell apoptosis and TNF. J Immunol.

[33] Andoh M, Zhang G, Russell-Lodrigue KE, Shive HR, Weeks BR, Samuel JE (2007). T cells are essential for bacterial clearance, and gamma interferon, tumor necrosis factor alpha, and B cells are crucial for disease development in Coxiella burnetii infection in mice. Infect Immun.

[34] Zvi A, Rotem S, Zauberman A, Elia U, Aftalion M, Bar-Haim E, et al. Novel CTL epitopes identified through a Y. pestis proteome-wide analysis in the search for vaccine candidates against plague. Vaccine. 2017;35(44):5995–6006. 10.1016/j.vaccine.2017.05.092 28606812

[35] Arricau-Bouvery N, Hauck Y, Bejaoui A, Frangoulidis D, Bodier CC, Souriau A (2006). Molecular characterization of Coxiella burnetii isolates by infrequent restriction site-PCR and MLVA typing. BMC Microbiol.

[36] Sekeyova Z, Roux V, Raoult D (1999). Intraspecies diversity of Coxiella burnetii as revealed by com1 and mucZ sequence comparison. FEMS Microbiol Lett.

[37] Hemsley CM, O'Neill PA, Essex-Lopresti A, Norville IH, Atkins TP, Titball RW (2019). Extensive genome analysis of Coxiella burnetii reveals limited evolution within genomic groups. BMC Genomics.

[38] Seshadri R, Paulsen IT, Eisen JA, Read TD, Nelson KE, Nelson WC (2003). Complete genome sequence of the Q-fever pathogen Coxiella burnetii. Proc Natl Acad Sci U S A.

[39] Long CM, Beare PA, Cockrell DC, Larson CL, Heinzen RA (2019). Comparative virulence of diverse Coxiella burnetii strains. Virulence.

[40] Ammerdorffer A, Kuley R, Dinkla A, Joosten LAB, Toman R, Roest HJ, et al. Coxiella burnetii isolates originating from infected cattle induce a more pronounced proinflammatory cytokine response compared to isolates from infected goats and sheep. Pathog Dis. 2017;75(4). 10.1093/femspd/ftx040 28387835

[41] Shpynov SN, Tarasevich IV, Skiba AA, Pozdnichenko NN, Gumenuk AS (2018). Comparison of genomes of Coxiella burnetii strains using formal order analysis. New Microbes New Infect.

[42] Pinero A, Barandika JF, Garcia-Perez AL, Hurtado A (2015). Genetic diversity and variation over time of Coxiella burnetii genotypes in dairy cattle and the farm environment. Infect Genet Evol.

[43] Pearson T, Hornstra HM, Hilsabeck R, Gates LT, Olivas SM, Birdsell DM, et al. High prevalence and two dominant host-specific genotypes of Coxiella burnetii in U.S. milk. BMC Microbiol. 2014;14:41. 10.1186/1471-2180-14-41 PMC3936997 24533573

[44] Glazunova O, Roux V, Freylikman O, Sekeyova Z, Fournous G, Tyczka J (2005). Coxiella burnetii genotyping. Emerg Infect Dis.

[45] Neumann RS, Kumar S, Haverkamp TH, Shalchian-Tabrizi K (2014). BLASTGrabber: a bioinformatic tool for visualization, analysis and sequence selection of massive BLAST data. BMC Bioinform.

[46] Maman Y, Nir-Paz R, Louzoun Y. Bacteria modulate the CD8+ T cell epitope repertoire of host cytosol-exposed proteins to manipulate the host immune response. PLoS Comput Biol. 2011;7(10):e1002220. 10.1371/journal.pcbi.1002220 PMC3192822 22022257

[47] Nielsen M, Connelley T, Ternette N (2018). Improved prediction of bovine leucocyte antigens (BoLA) presented ligands by use of mass-spectrometry-determined ligand and in vitro binding data. J Proteome Res.

[48] Rana A, Rub A, Akhter Y. Proteome-wide B and T cell epitope repertoires in outer membrane proteins of Mycobacterium avium subsp. paratuberculosis have vaccine and diagnostic relevance: a holistic approach. J Mol Recognit. 2015;28(8):506–20. 10.1002/jmr.2458 25727233

[49] Nain Z, Abdulla F, Rahman MM, Karim MM, Khan MSA, Sayed SB (2020). Proteome-wide screening for designing a multi-epitope vaccine against emerging pathogen Elizabethkingia anophelis using immunoinformatic approaches. J Biomol Struct Dyn.

[50] Ghasemi A, Ranjbar R, Amani J (2014). In silico analysis of chimeric TF, Omp31 and BP26 fragments of Brucella melitensis for development of a multi subunit vaccine candidate. Iran J Basic Med Sci.

[51] Wang L, Khattar MK, Donachie WD, Lutkenhaus J (1998). FtsI and FtsW are localized to the septum in *Escherichia coli*. J Bacteriol.

[52] Prachar M, Justesen S, Steen-Jensen DB, Thorgrimsen S, Jurgons E, Winther O (2020). Identification and validation of 174 COVID-19 vaccine candidate epitopes reveals low performance of common epitope prediction tools. Sci Rep.

[53] Ali A, Soares SC, Santos AR, Guimaraes LC, Barbosa E, Almeida SS (2012). Campylobacter fetus subspecies: comparative genomics and prediction of potential virulence targets. Gene.

[54] Jain S, Goldberg MB (2007). Requirement for YaeT in the outer membrane assembly of autotransporter proteins. J Bacteriol.

[55] Luedtke BE, Mahapatra S, Lutter EI, Shaw EI. The Coxiella Burnetii type IVB secretion system (T4BSS) component DotA is released/secreted during infection of host cells and during in vitro growth in a T4BSS-dependent manner. Pathog Dis. 2017;75(4). 10.1093/femspd/ftx047 PMC5437124 28449081

[56] Segal G, Feldman M, Zusman T (2005). The Icm/Dot type-IV secretion systems of Legionella pneumophila and Coxiella burnetii. FEMS Microbiol Rev.

[57] Wattam AR, Davis JJ, Assaf R, Boisvert S, Brettin T, Bun C (2017). Improvements to PATRIC, the all-bacterial Bioinformatics Database and Analysis Resource Center. Nucleic Acids Res.

[58] Pathosystems Resource Integration Center [Available from: https://www.patricbrc.org/.

[59] D'Amato F, Rouli L, Edouard S, Tyczka J, Million M, Robert C (2014). The genome of Coxiella burnetii Z3055, a clone linked to the Netherlands Q fever outbreaks, provides evidence for the role of drift in the emergence of epidemic clones. Comput Immunol Microbiol Infect Dis.

[60] Beare PA, Unsworth N, Andoh M, Voth DE, Omsland A, Gilk SD (2009). Comparative genomics reveal extensive transposon-mediated genomic plasticity and diversity among potential effector proteins within the genus Coxiella. Infect Immun.

[61] Kuley R, Kuijt E, Smits MA, Roest HIJ, Smith HE, Bossers A (2017). Genome plasticity and polymorphisms in critical genes correlate with increased virulence of dutch outbreak-related coxiella burnetii strains. Front Microbiol.

[62] Beare PA, Jeffrey BM, Martens CA, Pearson T, Heinzen RA. Draft Genome Sequences of Historical Strains of Coxiella burnetii Isolated from Cow's Milk and a Goat Placenta. Genome Announc. 2017;5(39). 10.1128/genomeA.00985-17 PMC5624756 28963210

[63] Ali A, Naz A, Soares SC, Bakhtiar M, Tiwari S, Hassan SS, et al. Pan-genome analysis of human gastric pathogen H. pylori: comparative genomics and pathogenomics approaches to identify regions associated with pathogenicity and prediction of potential core therapeutic targets. Biomed Res Int. 2015;2015:139580. 10.1155/2015/139580 PMC4325212 25705648

[64] McClain S. Bioinformatic screening and detection of allergen cross-reactive IgE-binding epitopes. Mol Nutr Food Res. 2017;61(8). 10.1002/mnfr.201600676 PMC5573986 28191711

[65] Gonzalez-Galarza FF, McCabe A, Santos E, Jones J, Takeshita L, Ortega-Rivera ND (2020). Allele frequency net database (AFND) 2020 update: gold-standard data classification, open access genotype data and new query tools. Nucleic Acids Res.

[66] Allele Frequency Net Database [Available from: http://www.allelefrequencies.net/default.asp.

[67] International Immunogenetics Information System/Human Leukocyte Antigen [Available from: https://www.ebi.ac.uk/ipd/imgt/hla/.

[68] Kumar S, Stecher G, Li M, Knyaz C, Tamura K (2018). MEGA X: Molecular Evolutionary Genetics Analysis across Computing Platforms. Mol Biol Evol.

[69] NetMHCpan 4.1 [Available from: https://services.healthtech.dtu.dk/service.php?NetMHCpan-4.1.

[70] NetMHCIIpan 4.0 [Available from: https://services.healthtech.dtu.dk/service.php?NetMHCIIpan-4.0.

[71] Perry AJ, Ho BK (2013). Inmembrane, a bioinformatic workflow for annotation of bacterial cell-surface proteomes. Source Code Biol Med.

[72] Juncker AS, Willenbrock H, Von Heijne G, Brunak S, Nielsen H, Krogh A (2003). Prediction of lipoprotein signal peptides in Gram-negative bacteria. Protein Sci.

[73] Krogh A, Larsson B, von Heijne G, Sonnhammer EL (2001). Predicting transmembrane protein topology with a hidden Markov model: application to complete genomes. J Mol Biol.

[74] Petersen TN, Brunak S, von Heijne G, Nielsen H. SignalP 4.0: discriminating signal peptides from transmembrane regions. Nat Methods. 2011;8(10):785–6. 10.1038/nmeth.1701 21959131

[75] Sigrist CJ, Cerutti L, Hulo N, Gattiker A, Falquet L, Pagni M (2002). PROSITE: a documented database using patterns and profiles as motif descriptors. Brief Bioinform.

