# Supplementary material for: Proteome-wide analysis of Coxiella burnetii for conserved T-cell epitopes with presentation across multiple host species
Source: BMC Bioinformatics. 2021 Jun 2;22:296. doi: 10.1186/s12859-021-04181-w (PMC8170629; doi:10.1186/s12859-021-04181-w)
Supplement: Supplementary file 2 — Additional file 2. Allelic phylogenetic analysis. Phylogenetic trees containing MHCII (A and B) or MHCI (C and D) alleles from human species. MHCII alleles were included based on geographical representation for the DRB1 locus or an allelic frequency of 0.05 or greater for the remaining loci. MHCI alleles were included based on geographical representation as denoted by AFND. 999 bootstraps were run during neighbor-joining tree generation for MHCII alleles (A), while 1,000 bootstraps were completed when producing the maximum likelihood tree for MHCII alleles (B). MHCI allelic comparison using either the neighbor-joining (C) or the maximum likelihood method (D) using 1,000 bootstraps. Trees were condensed to only show branching when bootstrap values were 80 or above. [file 12859_2021_4181_MOESM2_ESM.pdf]

Proteome-wide Analysis of *Coxiella burnetii* for Conserved T-cell epitopes with Presentation Across Multiple Host Species

Lindsay M.W. Piel[1], Codie J. Durfee[1], Stephen N. White[1,2,3]

[1] USDA-ARS Animal Disease Research Unit, Pullman, WA 99164, USA

[2] Department of Veterinary Microbiology & Pathology, Washington State University, Pullman, WA 99164, USA

[3] Center for Reproductive Biology, Washington State University, Pullman, WA 99164, USA

Correspondence: Stephen.White@usda.gov

A

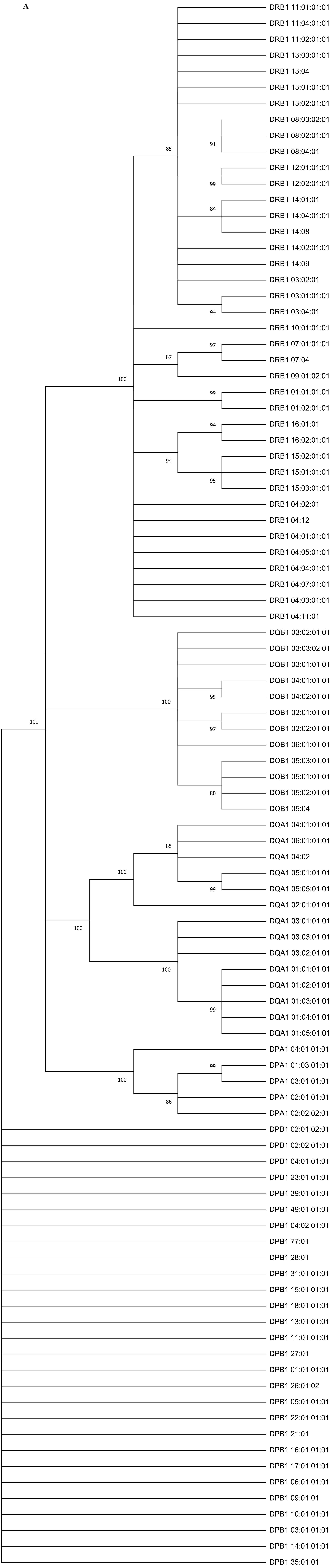
DRB1 11:01:01:01
DRB1 11:04:01:01
DRB1 11:02:01:01
DRB1 13:03:01:01
DRB1 13:04
DRB1 13:01:01:01
DRB1 13:02:01:01
DRB1 08:03:02:01
DRB1 08:02:01:01
DRB1 08:04:01
DRB1 12:01:01:01
DRB1 12:02:01:01
DRB1 14:01:01
DRB1 14:04:01:01
DRB1 14:08
DRB1 14:02:01:01
DRB1 14:09
DRB1 03:02:01
DRB1 03:01:01:01
DRB1 03:04:01
DRB1 10:01:01:01
DRB1 07:01:01:01
DRB1 07:04
DRB1 09:01:02:01
DRB1 01:01:01:01
DRB1 01:02:01:01
DRB1 16:01:01
DRB1 16:02:01:01
DRB1 15:02:01:01
DRB1 15:01:01:01
DRB1 15:03:01:01
DRB1 04:02:01
DRB1 04:12
DRB1 04:01:01:01
DRB1 04:05:01:01
DRB1 04:04:01:01
DRB1 04:07:01:01
DRB1 04:03:01:01
DRB1 04:11:01
DQB1 03:02:01:01
DQB1 03:03:02:01
DQB1 03:01:01:01
DQB1 04:01:01:01
DQB1 04:02:01:01
DQB1 02:01:01:01
DQB1 02:02:01:01
DQB1 06:01:01:01
DQB1 05:03:01:01
DQB1 05:01:01:01
DQB1 05:02:01:01
DQB1 05:04
DQA1 04:01:01:01
DQA1 06:01:01:01
DQA1 04:02
DQA1 05:01:01:01
DQA1 05:05:01:01
DQA1 02:01:01:01
DQA1 03:01:01:01
DQA1 03:03:01:01
DQA1 03:02:01:01
DQA1 01:01:01:01
DQA1 01:02:01:01
DQA1 01:03:01:01
DQA1 01:04:01:01
DQA1 01:05:01:01
DPA1 04:01:01:01
DPA1 01:03:01:01
DPA1 03:01:01:01
DPA1 02:01:01:01
DPA1 02:02:02:01
DPB1 02:01:02:01
DPB1 02:02:01:01
DPB1 04:01:01:01
DPB1 23:01:01:01
DPB1 39:01:01:01
DPB1 49:01:01:01
DPB1 04:02:01:01
DPB1 77:01
DPB1 28:01
DPB1 31:01:01:01
DPB1 15:01:01:01
DPB1 18:01:01:01
DPB1 13:01:01:01
DPB1 11:01:01:01
DPB1 27:01
DPB1 01:01:01:01
DPB1 26:01:02
DPB1 05:01:01:01
DPB1 22:01:01:01
DPB1 21:01
DPB1 16:01:01:01
DPB1 17:01:01:01
DPB1 06:01:01:01
DPB1 09:01:01
DPB1 10:01:01:01
DPB1 03:01:01:01
DPB1 14:01:01:01
DPB1 35:01:01

B

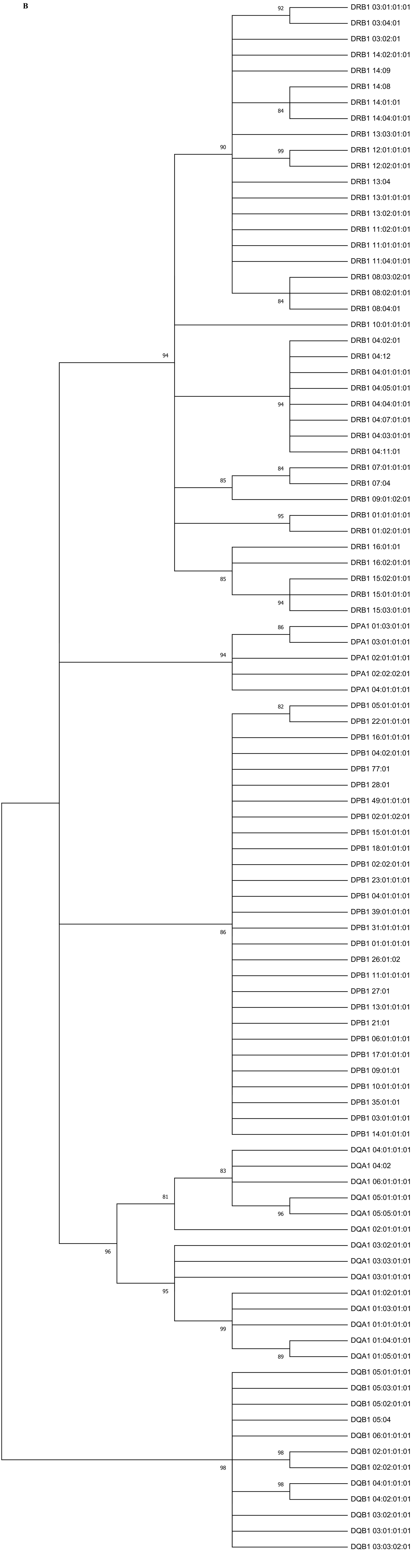
92
DRB1 03:01:01:01
DRB1 03:04:01
DRB1 03:02:01
DRB1 14:02:01:01
DRB1 14:09
DRB1 14:08
DRB1 14:01:01
84
DRB1 14:04:01:01
DRB1 13:03:01:01
90
99
DRB1 12:01:01:01
DRB1 12:02:01:01
DRB1 13:04
DRB1 13:01:01:01
DRB1 13:02:01:01
DRB1 11:02:01:01
DRB1 11:01:01:01
DRB1 11:04:01:01
DRB1 08:03:02:01
DRB1 08:02:01:01
84
DRB1 08:04:01
DRB1 10:01:01:01
DRB1 04:02:01
94
DRB1 04:12
DRB1 04:01:01:01
DRB1 04:05:01:01
DRB1 04:04:01:01
94
DRB1 04:07:01:01
DRB1 04:03:01:01
DRB1 04:11:01
84
DRB1 07:01:01:01
85
DRB1 07:04
DRB1 09:01:02:01
95
DRB1 01:01:01:01
DRB1 01:02:01:01
DRB1 16:01:01
DRB1 16:02:01:01
DRB1 15:02:01:01
85
DRB1 15:01:01:01
94
DRB1 15:03:01:01
86
DPA1 01:03:01:01
DPA1 03:01:01:01
94
DPA1 02:01:01:01
DPA1 02:02:02:01
DPA1 04:01:01:01
82
DPB1 05:01:01:01
DPB1 22:01:01:01
DPB1 16:01:01:01
DPB1 04:02:01:01
DPB1 77:01
DPB1 28:01
DPB1 49:01:01:01
DPB1 02:01:01:02
DPB1 15:01:01:01
DPB1 18:01:01:01
DPB1 02:02:01:01
DPB1 23:01:01:01
DPB1 04:01:01:01
DPB1 39:01:01:01
DPB1 31:01:01:01
86
DPB1 01:01:01:01
DPB1 26:01:02
DPB1 11:01:01:01
DPB1 27:01
DPB1 13:01:01:01
DPB1 21:01
DPB1 06:01:01:01
DPB1 17:01:01:01
DPB1 09:01:01
DPB1 10:01:01:01
DPB1 35:01:01
DPB1 03:01:01:01
DPB1 14:01:01:01
DQA1 04:01:01:01
DQA1 04:02
83
DQA1 06:01:01:01
81
DQA1 05:01:01:01
96
DQA1 05:05:01:01
DQA1 02:01:01:01
96
DQA1 03:02:01:01
DQA1 03:03:01:01
DQA1 03:01:01:01
95
DQA1 01:02:01:01
DQA1 01:03:01:01
99
DQA1 01:01:01:01
DQA1 01:04:01:01
89
DQA1 01:05:01:01
DQB1 05:01:01:01
DQB1 05:03:01:01
DQB1 05:02:01:01
DQB1 05:04
DQB1 06:01:01:01
98
DQB1 02:01:01:01
98
DQB1 02:02:01:01
98
DQB1 04:01:01:01
DQB1 04:02:01:01
DQB1 03:02:01:01
DQB1 03:01:01:01
DQB1 03:03:02:01

C

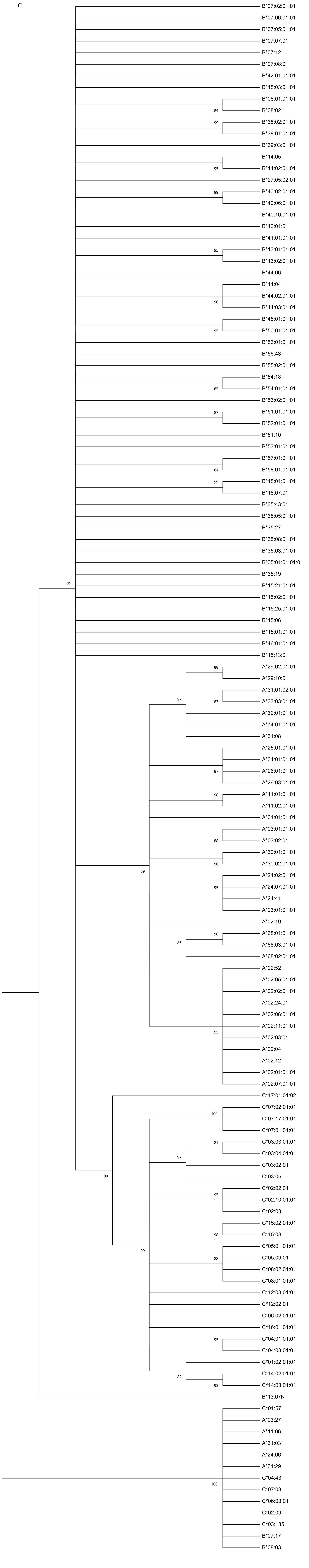
B*07:02:01:01
B*07:06:01:01
B*07:05:01:01
B*07:07:01
B*07:12
B*07:08:01
B*42:01:01:01
B*48:03:01:01
B*08:01:01:01
84
B*08:02
99
B*38:02:01:01
B*38:01:01:01
B*39:03:01:01
B*14:05
95
B*14:02:01:01
B*27:05:02:01
99
B*40:02:01:01
B*40:06:01:01
B*40:10:01:01
B*40:01:01
B*41:01:01:01
95
B*13:01:01:01
B*13:02:01:01
B*44:06
B*44:04
B*44:02:01:01
90
B*44:03:01:01
B*45:01:01:01
95
B*50:01:01:01
B*56:01:01:01
B*56:43
B*55:02:01:01
B*54:18
85
B*54:01:01:01
B*56:02:01:01
87
B*51:01:01:01
B*52:01:01:01
B*51:10
B*53:01:01:01
B*57:01:01:01
84
B*58:01:01:01
99
B*18:01:01:01
B*18:07:01
B*35:43:01
B*35:05:01:01
B*35:27
B*35:08:01:01
B*35:03:01:01
B*35:01:01:01:01
B*35:19
99
B*15:21:01:01
B*15:02:01:01
B*15:25:01:01
B*15:06
B*15:01:01:01
B*46:01:01:01
B*15:13:01
99
A*29:02:01:01
A*29:10:01
87
A*31:01:02:01
83
A*33:03:01:01
A*32:01:01:01
A*74:01:01:01
A*31:08
A*25:01:01:01
A*34:01:01:01
87
A*26:01:01:01
A*26:03:01:01
98
A*11:01:01:01
A*11:02:01:01
A*01:01:01:01
A*03:01:01:01
88
A*03:02:01
A*30:01:01:01
90
A*30:02:01:01
89
A*24:02:01:01
A*24:07:01:01
95
A*24:41
A*23:01:01:01
A*02:19
98
A*68:01:01:01
85
A*68:03:01:01
A*68:02:01:01
A*02:52
A*02:05:01:01
A*02:02:01:01
A*02:24:01
A*02:06:01:01
A*02:11:01:01
95
A*02:03:01
A*02:04
A*02:12
A*02:01:01:01
A*02:07:01:01
C*17:01:01:02
100
C*07:02:01:01
C*07:17:01:01
C*07:01:01:01
81
C*03:03:01:01
C*03:04:01:01
97
C*03:02:01
80
C*03:05
C*02:02:01
95
C*02:10:01:01
C*02:03
C*15:02:01:01
98
C*15:03
99
C*05:01:01:01
88
C*05:09:01
C*08:02:01:01
C*08:01:01:01
C*12:03:01:01
C*12:02:01
C*06:02:01:01
C*16:01:01:01
95
C*04:01:01:01
C*04:03:01:01
C*01:02:01:01
82
C*14:02:01:01
93
C*14:03:01:01
B*13:07N
C*01:57
A*03:27
A*11:06
A*31:03
A*24:06
A*31:29
C*04:43
100
C*07:03
C*06:03:01
C*02:09
C*03:135
B*07:17
B*08:03

D

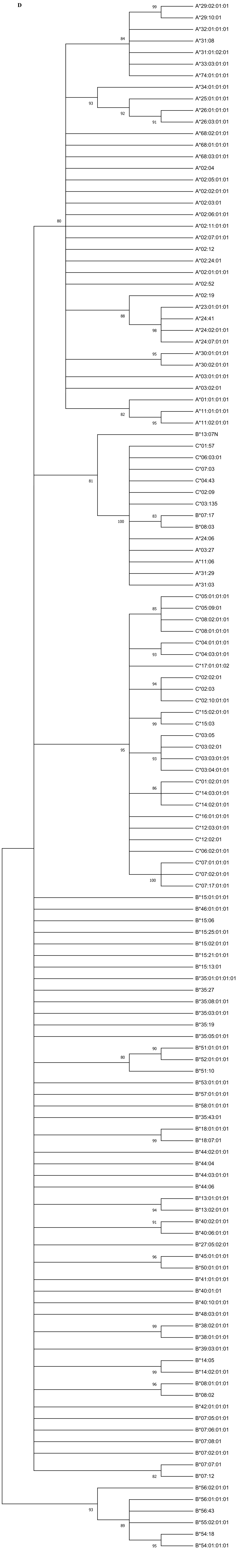
A*29:02:01:01
A*29:10:01
A*32:01:01:01
A*31:08
A*31:01:02:01
A*33:03:01:01
A*74:01:01:01
A*34:01:01:01
A*25:01:01:01
A*26:01:01:01
A*26:03:01:01
A*68:02:01:01
A*68:01:01:01
A*68:03:01:01
A*02:04
A*02:05:01:01
A*02:02:01:01
A*02:03:01
A*02:06:01:01
A*02:11:01:01
A*02:07:01:01
A*02:12
A*02:24:01
A*02:01:01:01
A*02:52
A*02:19
A*23:01:01:01
A*24:41
A*24:02:01:01
A*24:07:01:01
A*30:01:01:01
A*30:02:01:01
A*03:01:01:01
A*03:02:01
A*01:01:01:01
A*11:01:01:01
A*11:02:01:01
B*13:07N
C*01:57
C*06:03:01
C*07:03
C*04:43
C*02:09
C*03:135
B*07:17
B*08:03
A*24:06
A*03:27
A*11:06
A*31:29
A*31:03
C*05:01:01:01
C*05:09:01
C*08:02:01:01
C*08:01:01:01
C*04:01:01:01
C*04:03:01:01
C*17:01:01:02
C*02:02:01
C*02:03
C*02:10:01:01
C*15:02:01:01
C*15:03
C*03:05
C*03:02:01
C*03:03:01:01
C*03:04:01:01
C*01:02:01:01
C*14:03:01:01
C*14:02:01:01
C*16:01:01:01
C*12:03:01:01
C*12:02:01
C*06:02:01:01
C*07:01:01:01
C*07:02:01:01
C*07:17:01:01
B*15:01:01:01
B*46:01:01:01
B*15:06
B*15:25:01:01
B*15:02:01:01
B*15:21:01:01
B*15:13:01
B*35:01:01:01:01
B*35:27
B*35:08:01:01
B*35:03:01:01
B*35:19
B*35:05:01:01
B*51:01:01:01
B*52:01:01:01
B*51:10
B*53:01:01:01
B*57:01:01:01
B*58:01:01:01
B*35:43:01
B*18:01:01:01
B*18:07:01
B*44:02:01:01
B*44:04
B*44:03:01:01
B*44:06
B*13:01:01:01
B*13:02:01:01
B*40:02:01:01
B*40:06:01:01
B*27:05:02:01
B*45:01:01:01
B*50:01:01:01
B*41:01:01:01
B*40:01:01
B*40:10:01:01
B*48:03:01:01
B*38:02:01:01
B*38:01:01:01
B*39:03:01:01
B*14:05
B*14:02:01:01
B*08:01:01:01
B*08:02
B*42:01:01:01
B*07:05:01:01
B*07:06:01:01
B*07:08:01
B*07:02:01:01
B*07:07:01
B*07:12
B*56:02:01:01
B*56:01:01:01
B*56:43
B*55:02:01:01
B*54:18
B*54:01:01:01
