# Supplementary material for: Proteome-wide analysis of Coxiella burnetii for conserved T-cell epitopes with presentation across multiple host species
Source: BMC Bioinformatics. 2021 Jun 2;22:296. doi: 10.1186/s12859-021-04181-w (PMC8170629; doi:10.1186/s12859-021-04181-w)
Supplement: Supplementary file 3 — Additional file 3. Isolation of quality controlled MHCII and MHCI epitopes. Contains the methodology used to select output epitopes of interest. [file 12859_2021_4181_MOESM3_ESM.docx]

Proteome-wide Analysis of *Coxiella burnetii* for Conserved T-cell epitopes with Presentation Across Multiple Host Species

Lindsay M.W. Piel^1^, Codie J. Durfee^1^, Stephen N. White^1,2,3^

^1^ USDA-ARS Animal Disease Research Unit, Pullman, WA 99164, USA

^2^ Department of Veterinary Microbiology & Pathology, Washington State University, Pullman, WA 99164, USA

^3^ Center for Reproductive Biology, Washington State University, Pullman, WA 99164, USA

Correspondence: Stephen.White@usda.gov

**Filtering datasets for qualitative epitopes.** Previously studied epitopes from human, murine, or caprine studies are found in Additional Table 1. To determine the highest quality epitopes, the resultant NetMHCpan data was examined for matching proteins and epitope type in Additional Table 1. Notably, thirteen of the previously studied proteins were removed due to a lack of conservation between *Coxiella burnetii* isolates or from the presence of host homology. These protein locus tags are denoted by an asterisk in Additional Table 1. Of the remaining 21 proteins, the average number of alleles bound or strong allelic interactions was calculated per species of interest. This average was employed to ascertain the best cut-off values for filtering the raw data output.
