# Supplementary material for: Proteome-wide analysis of Coxiella burnetii for conserved T-cell epitopes with presentation across multiple host species
Source: BMC Bioinformatics. 2021 Jun 2;22:296. doi: 10.1186/s12859-021-04181-w (PMC8170629; doi:10.1186/s12859-021-04181-w)
Supplement: Supplementary file 4 — Additional file 4. Previously studied Coxiella burnetii epitopes. Locus tag and gene name based upon genomic assembly annotations for Nine Mile Phase I (RSA 493) on National Center for Biotechnology and Information (NCBI). Species indicates which host or model organism the epitope was analyzed for. The epitope type column describes if the peptide studied was in regards to a B-cell or T-cell (MHCI or MHCII) epitope. If more than one epitope was isolated, then the epitope types are separated by backslashes to indicate the order of MHC epitopes or an ampersand to indicate B-cell production of antibodies. Locus tag superscripts denote protein subcellular location and if the protein was disqualified from NetMHCpan due to previous analysis. 1 is for membrane associated, 2 is for cytoplasmic location, 3 is for unknown location, and an asterisk indicates removal. Epitope amino acid positions are annotated to represent the pre-processed forms of the proteins [file 12859_2021_4181_MOESM4_ESM.docx]

Proteome-wide Analysis of *Coxiella burnetii* for Conserved T-cell epitopes with Presentation Across Multiple Host Species

Lindsay M.W. Piel^1^, Codie J. Durfee^1^, Stephen N. White^1,2,3^

^1^ USDA-ARS Animal Disease Research Unit, Pullman, WA 99164, USA

^2^ Department of Veterinary Microbiology & Pathology, Washington State University, Pullman, WA 99164, USA

^3^ Center for Reproductive Biology, Washington State University, Pullman, WA 99164, USA

Correspondence: Stephen.White@usda.gov

| **Locus Tag (Gene Name)** | **Species** | **Epitopes Found** | **Experimental Process** | **Epitope**  **Type** | **Ref.** |
| --- | --- | --- | --- | --- | --- |
| **CBU_0077^1^** | Human | 203-LTLLLNWVNY-212 | Bioinformatic Screening with IFN-γ Recall Production | MHCI | (1) |
| **CBU_0092^1^**  **(*ybgF*)** | Murine | 185-LLTKKQYDKAQASFQ-199 | Immunologic Response in Immunized Mice | MHCII | (2) |
|  | Human |  | Serology against Protein Microarray | B-cell | (3) |
|  | Human | 196-ASFQNYLNDY-205 | Bioinformatic Screening with IFN-γ Recall Production | MHCI | (1) |
| **CBU_0109^1*^** | Human |  | Serology against Protein Microarray | B-cell | (3) |
|  | Human | 247-SPAVLSAAKKIFGDGA-262  105-KTFVYPMGLY-114 | Bioinformatic Screening with IFN-γ Recall Production | MHCII/I | (1) |
| **CBU_0307^1^** | Murine | 146-GKLGVAYTYNRANAG-160 | Immunologic Response in Immunized Mice | MHCII | (2) |
|  | Human |  | Serology against Protein Microarray | B-cell | (4) |
|  | Human | 149-GVAYTYNRANAGLPTNK-165  202-VPGYRNASSKRFVAP-216 | Bioinformatic Screening with IFN-γ Recall Production | MHCII | (1) |
|  | Goat &  Human |  | Meta-analysis with Goat and Human Serology Testing | B-cell | (5) |
| **CBU_0311^1^** | Murine | 70-PVSASITQFGPVGEL-84 | Immunologic Response in Immunized Mice | MHCII | (2) |
|  | Murine |  | IFN-γ and Antibody Production | B-cell | (6) |
| **CBU_0545^1^**  **(*lemA*)** | Human |  | Serology against Protein Microarray | B-cell | (3, 4) |
| **CBU_0612^1^**  **(*ompH*)** | Human | 41-IKDINTRLEK-50  76-DEAVMGKKEAENLR-89  90-KEIQNDESTLRQQQQQ-105  123-SKVNGAVKRVAE-134 | Bioinformatic Analysis | MHCI/I/II/I | (7) |
|  | Murine | 13-VAMIWSVAAVAQTVG-27 | Immunologic Response in Immunized Mice | MHCII | (2) |
|  | Human |  | Serology against Protein Microarray | B-cell | (3, 4) |
| **CBU_0630^1*^**  **(*fkpA*)** | Murine | 159-FDSSYKRGQPATFPL-173 | Immunologic Response in Immunized Mice | MHCII | (2) |
|  | Murine |  | Protection from *C. burnetii* Infection using Antigen Stimulated BMDC | MHCII | (8) |
|  | Human |  | Serology against Protein Microarray | B-cell | (3) |
| **CBU_0718^1^** | Human | 21-VAKLRGDLSSIIHKL-35  28-LSSIIHKLTSFSKTEA-43 | Bioinformatic Screening with IFN-γ Recall Production | MHCII | (1) |
|  | Goat &  Human |  | Meta-analysis with Goat and Human Serology Testing | B-cell | (5) |
| **CBU_0754^1^** | Human |  | Serology against Protein Microarray | B-cell | (4) |
| **CBU_0891^1^** | Human |  | Serology against Protein Microarray | B-cell | (3, 4) |
|  | Goat &  Human |  | Meta-analysis with Goat and Human Serology Testing | B-cell | (5) |
| **CBU_0968^1*^** | Human | 144-AENVLIIHNKTLAHRYLA-161 | Bioinformatic Screening with IFN-γ Recall Production | MHCII | (1) |
| **CBU_1143^1^**  **(*yajC*)** | Human |  | Serology against Protein Microarray | B-cell | (3, 4) |
|  | Human | 94-GTEITVQKASIASVLPK-110 | Bioinformatic Screening with IFN-γ Recall Production | MHCII | (1) |
| **CBU_1157^1^** | Murine | 88-PWRYIRSFPILASSG-102  133-LSLMLNYPNSADRYY-147  202-DLRYHAPIYGAVHPR-216 | IFN-γ and Antibody Production | MHCII&  B-cell | (6) |
|  | Human | 130-RFDLSLMLNYPNSADRY-146 | Bioinformatic Screening with IFN-γ Recall Production | MHCII | (1) |
| **CBU_1260^1^** | Human | 178-LPPVTSSVAVKVPSS-192 | Bioinformatic Screening with IFN-γ Recall Production | MHCII | (1) |
| **CBU_1853^1*^** | Human |  | Serology against Protein Microarray | B-cell | (4) |
| **CBU_1869^1^** | Human |  | Serology against Protein Microarray | B-cell | (4) |
|  | Human | 200-GKHFDGIKVLKLSPQNTI-217 | Bioinformatic Screening with IFN-γ Recall Production | MHCII | (1) |
|  | Murine |  | IFN-γ and Antibody Production | B-cell | (6) |
| **CBU_1910^1^**  **(*com1*)** | Human | 59-QKKTEAQQEEHAQQAIKEN-77  121-VKQNKNLRV-129  214-QLAGTPTFVI-223 | Bioinformatic Analysis | MHCII/I/I | (7) |
|  | Murine | 42-HYLVNHPEVLVEASQ-56 | Immunologic Response in Immunized Mice | MHCII | (2) |
|  | Murine |  | Protection from *C. burnetii* Infection using Antigen Stimulated BMDC | MHCII | (8) |
|  | Murine | 42-HYLVNHPEVLVEASQ-56  80-KLFNDPASPVAGNPH-94 | IFN-γ and Antibody Production | MHCII &  B-cell | (6) |
|  | Human |  | Serology against Protein Microarray | B-cell | (3) |
|  | Human | 218-TPTFVIGNKALTKFGF-233  97-VTLVEFFDY-105  34-KDIQSIVHHY-43 | Bioinformatic Screening with IFN-γ Recall Production | MHCII/I/I | (1) |
| **CBU_1943^1*^**  **(*atpA*)** | Goat &  Human |  | Meta-analysis with Goat and Human Serology Testing | B-cell | (5) |
| **CBU_1967^1^** | Human |  | Serology against Protein Microarray | B-cell | (4) |
| **CBU_2065^1^** | Human |  | Serology against Protein Microarray | B-cell | (4) |
| **CBU_0229^2*^**  **(*rplL*)** | Human |  | Serology against Protein Microarray | B-cell | (3) |
|  | Human | 71-KIGVIKAIRTITGLGLKEA-89 | Bioinformatic Screening with IFN-γ Recall Production | MHCII | (1) |
| **CBU_0383^2^**  **(*tag*)** | Murine | 74-RDSFNNFDASIISKY-88 | IFN-γ and Antibody Production | MHCII&  B-cell | (6) |
|  | Goat &  Human |  | Meta-analysis with Goat and Human Serology Testing | B-cell | (5) |
| **CBU_1200^2^**  **(*icd*)** | Human | 139-LRPVRYFTGVPSPVKTPE-156 | Bioinformatic Screening with IFN-γ Recall Production | MHCII | (1) |
| **CBU_1398^2*^**  **(*sucB*)** | Human |  | Serology against Protein Microarray | B-cell | (3, 4) |
|  | Human | 231-RLGFMSFFTKAVVEALKRF-249  382-REAVLFLVTIKELLEDP-398 | Bioinformatic Screening with IFN-γ Recall Production | MHCII | (1) |
|  | Goat &  Human |  | Meta-analysis with Goat and Human Serology Testing | B-cell | (5) |
| **CBU_1416^2^** | Human | 74-IARYFMVNISQLIGEE-89 | Bioinformatic Screening with IFN-γ Recall Production | MHCII | (1) |
| **CBU_1513^2*^** | Human |  | Serology against Protein Microarray | B-cell | (3) |
|  | Human | 137-QGHIINIGSISSHQV-151  208-EAVYKGFTPLKAEDIAEA-225 | Bioinformatic Screening with IFN-γ Recall Production | MHCII | (1) |
|  | Goat &  Human |  | Meta-analysis with Goat and Human Serology Testing | B-cell | (5) |
| **CBU_1645^2^**  **(dotB)** | Murine | 193-YDSLTTPTASVCQSE-207  269-RLVGSFPAEERIGRT-283 | IFN-γ and Antibody Production | MHCII &  B-cell | (6) |
| **CBU_1706^2*^** | Goat &  Human |  | Meta-analysis with Goat and Human Serology Testing | B-cell | (5) |
| **CBU_1716^2*^**  **(gcvT)** | Human | 373-KIPVKIIKPPFVRRG-387 | Bioinformatic Screening with IFN-γ Recall Production | MHCII | (1) |
| **CBU_1718^2*^**  **(groEL)** | Murine | 474-DVNYGYNAATGEYGD-488 | Immunologic Response in Immunized Mice | MHCII | (2) |
|  | Goat &  Human |  | Meta-analysis with Goat and Human Serology Testing | B-cell | (5) |
| **CBU_1719^2*^**  **(groES)** | Human |  | Serology against Protein Microarray | B-cell | (3) |
| **CBU_1835^2^** | Human | 291-PDYVLNAVNHIRYKP-305  416-MMEHLQNITNLVSTGRQGA-434 | Bioinformatic Screening with IFN-γ Recall Production | MHCII | (1) |
| **CBU_0008^3*^** | Murine |  | IFN-γ and Antibody Production | B-cell | (6) |

1. Scholzen A, Richard G, Moise L, Baeten LA, Reeves PM, Martin WD, et al. Promiscuous Coxiella burnetii CD4 Epitope Clusters Associated With Human Recall Responses Are Candidates for a Novel T-Cell Targeted Multi-Epitope Q Fever Vaccine. Front Immunol. 2019;10:207.

2. Xiong X, Qi Y, Jiao J, Gong W, Duan C, Wen B. Exploratory study on Th1 epitope-induced protective immunity against Coxiella burnetii infection. PLoS One. 2014;9(1):e87206.

3. Vigil A, Ortega R, Nakajima-Sasaki R, Pablo J, Molina DM, Chao CC, et al. Genome-wide profiling of humoral immune response to Coxiella burnetii infection by protein microarray. Proteomics. 2010;10(12):2259-69.

4. Beare PA, Chen C, Bouman T, Pablo J, Unal B, Cockrell DC, et al. Candidate antigens for Q fever serodiagnosis revealed by immunoscreening of a Coxiella burnetii protein microarray. Clin Vaccine Immunol. 2008;15(12):1771-9.

5. Miller HK, Kersh GJ. Analysis of recombinant proteins for Q fever diagnostics. Sci Rep. 2020;10(1):20934.

6. Chen C, Dow C, Wang P, Sidney J, Read A, Harmsen A, et al. Identification of CD4+ T cell epitopes in C. burnetii antigens targeted by antibody responses. PLoS One. 2011;6(3):e17712.

7. Jaydari A, Forouharmehr A, Nazifi N. Determination of immunodominant scaffolds of Com1 and OmpH antigens of Coxiella burnetii. Microb Pathog. 2019;126:298-309.

8. Xiong X, Meng Y, Wang X, Qi Y, Li J, Duan C, et al. Mice immunized with bone marrow-derived dendritic cells stimulated with recombinant Coxiella burnetii Com1 and Mip demonstrate enhanced bacterial clearance in association with a Th1 immune response. Vaccine. 2012;30(48):6809-15.
