# Supplementary material for: Proteome-wide analysis of Coxiella burnetii for conserved T-cell epitopes with presentation across multiple host species
Source: BMC Bioinformatics. 2021 Jun 2;22:296. doi: 10.1186/s12859-021-04181-w (PMC8170629; doi:10.1186/s12859-021-04181-w)
Supplement: Supplementary file 11 — Additional file 11. MHCI and MHCII tested alleles. (A) MHCI alleles tested during the use of NetMHCpan 4.1. Human, murine, and bovine alleles are notated HLA, H-2, and BoLA respectively. (B) MHCII alleles tested during exploitation of NetMHCIIpan 4.0. Murine and human alleles are designated by H-2 or HLA respectively. Notably, the human DRA1 locus is not highly variable, therefore only the DRB1 allele for this pairing changed. Otherwise, each of the DPA1 and DQA1 loci were paired and tested with each of their respective DPB1 and DQB1 loci. [file 12859_2021_4181_MOESM11_ESM.docx]

Proteome-wide Analysis of *Coxiella burnetii* for Conserved T-cell epitopes with Presentation Across Multiple Host Species

Lindsay M.W. Piel^1^, Codie J. Durfee^1^, Stephen N. White^1,2,3^

^1^ USDA-ARS Animal Disease Research Unit, Pullman, WA 99164, USA

^2^ Department of Veterinary Microbiology & Pathology, Washington State University, Pullman, WA 99164, USA

^3^ Center for Reproductive Biology, Washington State University, Pullman, WA 99164, USA

Correspondence: Stephen.White@usda.gov

**Additional Table 8A.**

| **MHCI Alleles** | | | | |
| --- | --- | --- | --- | --- |
| **Bovine** | | **Murine** | **Human** | |
| BoLA-1:00901 | BoLA-3:00402 | H-2-Db | HLA-A01:01 | HLA-B35:01 |
| BoLA-1:00902 | BoLA-3:00403 | H-2-Dd | HLA-A02:01 | HLA-B35:03 |
| BoLA-1:01901 | BoLA-3:01001 | H-2-Kb | HLA-A02:02 | HLA-B35:05 |
| BoLA-1:02001 | BoLA-3:01101 | H-2-Kd | HLA-A02:03 | HLA-B35:08 |
| BoLA-1:02101 | BoLA-3:01701 | H-2-Kk | HLA-A02:04 | HLA-B35:19 |
| BoLA-1:02301 | BoLA-3:01702 | H-2-Ld | HLA-A02:05 | HLA-B35:27 |
| BoLA-1:02801 | BoLA-3:01703 | H-2-Qa1 | HLA-A02:06 | HLA-B35:43 |
| BoLA-1:02901 | BoLA-3:02701 | H-2-Qa2 | HLA-A02:07 | HLA-B38:01 |
| BoLA-1:03101 | BoLA-3:02702 |  | HLA-A02:11 | HLA-B39:03 |
| BoLA-1:03102 | BoLA-3:03501 |  | HLA-A02:12 | HLA-B40:01 |
| BoLA-1:04201 | BoLA-3:03601 |  | HLA-A02:19 | HLA-B40:10 |
| BoLA-1:04901 | BoLA-3:03701 |  | HLA-A02:24 | HLA-B41:01 |
| BoLA-1:06101 | BoLA-3:03801 |  | HLA-A02:52 | HLA-B42:01 |
| BoLA-1:06701 | BoLA-3:05001 |  | HLA-A03:01 | HLA-B44:02 |
| BoLA-1:07401 | BoLA-3:05002 |  | HLA-A11:01 | HLA-B44:03 |
| BoLA-2:00501 | BoLA-3:05101 |  | HLA-A24:02 | HLA-B45:01 |
| BoLA-2:00601 | BoLA-3:05201 |  | HLA-A26:01 | HLA-B46:01 |
| BoLA-2:00602 | BoLA-3:05301 |  | HLA-A29:02 | HLA-B48:03 |
| BoLA-2:00801 | BoLA-3:05801 |  | HLA-A30:01 | HLA-B51:01 |
| BoLA-2:00802 | BoLA-3:05901 |  | HLA-A31:08 | HLA-B51:10 |
| BoLA-2:01201 | BoLA-3:06501 |  | HLA-A32:01 | HLA-B53:01 |
| BoLA-2:01601 | BoLA-3:06601 |  | HLA-A33:03 | HLA-B54:01 |
| BoLA-2:01602 | BoLA-3:06602 |  | HLA-A68:01 | HLA-B56:01 |
| BoLA-2:01801 | BoLA-3:06801 |  | HLA-A68:02 | HLA-B56:02 |
| BoLA-2:01802 | BoLA-3:07301 |  | HLA-A74:01 | HLA-B57:01 |
| BoLA-2:02201 | BoLA-4:02401 |  | HLA-B07:02 | HLA-C01:02 |
| BoLA-2:02501 | BoLA-4:02402 |  | HLA-B07:05 | HLA-C01:57 |
| BoLA-2:02601 | BoLA-4:06301 |  | HLA-B07:07 | HLA-C02:02 |
| BoLA-2:02602 | BoLA-5:00301 |  | HLA-B07:08 | HLA-C03:02 |
| BoLA-2:02603 | BoLA-5:03901 |  | HLA-B07:12 | HLA-C03:04 |
| BoLA-2:03001 | BoLA-5:06401 |  | HLA-B08:01 | HLA-C03:05 |
| BoLA-2:03202 | BoLA-5:07201 |  | HLA-B13:01 | HLA-C04:01 |
| BoLA-2:04301 | BoLA-6:01301 |  | HLA-B14:02 | HLA-C05:01 |
| BoLA-2:04401 | BoLA-6:01302 |  | HLA-B15:01 | HLA-C06:02 |
| BoLA-2:04402 | BoLA-6:01401 |  | HLA-B15:02 | HLA-C07:02 |
| BoLA-2:04501 | BoLA-6:01402 |  | HLA-B15:06 | HLA-C12:02 |
| BoLA-2:04601 | BoLA-6:01501 |  | HLA-B15:13 | HLA-C12:03 |
| BoLA-2:04701 | BoLA-6:01502 |  | HLA-B15:21 | HLA-C14:02 |
| BoLA-2:04801 | BoLA-6:03401 |  | HLA-B15:25 | HLA-C15:02 |
| BoLA-2:05401 | BoLA-6:04001 |  | HLA-B18:01 | HLA-C16:01 |
| BoLA-2:05501 | BoLA-6:04101 |  | HLA-B27:05 | HLA-C17:01 |
| BoLA-2:05601 | BoLA-amani.1 |  |  |  |
| BoLA-2:05701 | BoLA-AW10 |  |  |  |
| BoLA-2:06001 | BoLA-D18.4 |  |  |  |
| BoLA-2:06201 | BoLA-gb1.7 |  |  |  |
| BoLA-2:06901 | BoLA-HD6 |  |  |  |
| BoLA-2:07001 | BoLA-JSP.1 |  |  |  |
| BoLA-2:07101 | BoLA-T2a |  |  |  |
| BoLA-3:00101 | BoLA-T2b |  |  |  |
| BoLA-3:00102 | BoLA-T2c |  |  |  |
| BoLA-3:00103 | BoLA-T5 |  |  |  |
| BoLA-3:00201 | BoLA-T7 |  |  |  |
| BoLA-3:00401 |  |  |  |  |

**Additional Table 8B.**

| **MHCII Alleles** | | | | | |
| --- | --- | --- | --- | --- | --- |
| **Murine** | **Human** | | | | |
| **H-2 locus** | **DRB1 locus** | **DPA1 locus** | **DPB1 locus** | **DQA1 locus** | **DQB1 locus** |
| H-2-IAb | HLA-DRB101:01 | HLA-DPA101:03 | HLA-DPB101:01 | HLA-DQA101:02 | HLA-DQB102:01 |
| H-2-IAd | HLA-DRB103:01 | HLA-DPA102:01 | HLA-DPB102:01 | HLA-DQA101:04 | HLA-DQB103:01 |
| H-2-IAk | HLA-DRB103:02 | HLA-DPA102:02 | HLA-DPB102:02 | HLA-DQA102:01 | HLA-DQB103:02 |
| H-2-IAq | HLA-DRB104:01 | HLA-DPA104:01 | HLA-DPB103:01 | HLA-DQA103:01 | HLA-DQB103:03 |
| H-2-IAs | HLA-DRB104:02 |  | HLA-DPB104:01 | HLA-DQA103:02 | HLA-DQB104:02 |
| H-2-IAu | HLA-DRB104:03 |  | HLA-DPB104:02 | HLA-DQA103:03 | HLA-DQB105:02 |
| H-2-IEd | HLA-DRB104:04 |  | HLA-DPB105:01 | HLA-DQA104:01 | HLA-DQB106:01 |
| H-2-IEk | HLA-DRB104:05 |  | HLA-DPB106:01 | HLA-DQA104:02 |  |
|  | HLA-DRB104:07 |  | HLA-DPB109:01 | HLA-DQA105:01 |  |
|  | HLA-DRB104:11 |  | HLA-DPB110:01 | HLA-DQA106:01 |  |
|  | HLA-DRB104:12 |  | HLA-DPB111:01 |  |  |
|  | HLA-DRB107:01 |  | HLA-DPB113:01 |  |  |
|  | HLA-DRB108:03 |  | HLA-DPB114:01 |  |  |
|  | HLA-DRB109:01 |  | HLA-DPB115:01 |  |  |
|  | HLA-DRB110:01 |  | HLA-DPB116:01 |  |  |
|  | HLA-DRB111:01 |  | HLA-DPB117:01 |  |  |
|  | HLA-DRB111:02 |  | HLA-DPB118:01 |  |  |
|  | HLA-DRB111:04 |  | HLA-DPB121:01 |  |  |
|  | HLA-DRB112:01 |  | HLA-DPB123:01 |  |  |
|  | HLA-DRB113:01 |  | HLA-DPB127:01 |  |  |
|  | HLA-DRB113:02 |  | HLA-DPB126:01 |  |  |
|  | HLA-DRB113:03 |  | HLA-DPB128:01 |  |  |
|  | HLA-DRB113:04 |  | HLA-DPB131:01 |  |  |
|  | HLA-DRB114:01 |  | HLA-DPB135:01 |  |  |
|  | HLA-DRB114:02 |  | HLA-DPB139:01 |  |  |
|  | HLA-DRB114:09 |  | HLA-DPB149:01 |  |  |
|  | HLA-DRB115:01 |  | HLA-DPB177:01 |  |  |
|  | HLA-DRB116:02 |  |  |  |  |
