# Supplementary material for: Proteome-wide analysis of Coxiella burnetii for conserved T-cell epitopes with presentation across multiple host species
Source: BMC Bioinformatics. 2021 Jun 2;22:296. doi: 10.1186/s12859-021-04181-w (PMC8170629; doi:10.1186/s12859-021-04181-w)
Supplement: Supplementary file 12 — Additional file 12. Exclusion of Dugway 5J108-111. (A) Protein GenBank IDs returned to analysis when Dugway 5J108-111 was removed from inter-isolate comparison. Homology to host species is noted in the second column, where a yes indicates removal of the protein before T-cell epitope analysis. (B) C. burnetii defined MHCI T-cell epitopes represented within human, murine, and bovine species during Dugway 5J108-111 exclusion. Pos indicates the position at which the peptide begins within the protein of interest. GenBank ID, gene name, and locus tag provide protein identification parameters present in assembly ASM776v2. Protein localization was defined through the use of Inmembrane. Program updates labeled the location of AAO91013.1 as IM+peri (inner membrane plus the periplasmic space), this was altered to Membrane (non-PSE) to keep with location labels in the remainder of the manuscript. [file 12859_2021_4181_MOESM12_ESM.docx]

Proteome-wide Analysis of *Coxiella burnetii* for Conserved T-cell epitopes with Presentation Across Multiple Host Species

Lindsay M.W. Piel^1^, Codie J. Durfee^1^, Stephen N. White^1,2,3^

^1^ USDA-ARS Animal Disease Research Unit, Pullman, WA 99164, USA

^2^ Department of Veterinary Microbiology & Pathology, Washington State University, Pullman, WA 99164, USA

^3^ Center for Reproductive Biology, Washington State University, Pullman, WA 99164, USA

Correspondence: Stephen.White@usda.gov

(A)

| **GenBank ID** | **Host Homology** |
| --- | --- |
| AAO89588.1 | No |
| AAO89598.2 | Yes |
| AAO89778.1 | No |
| AAO89876.1 | No |
| AAO90298.1 | Yes |
| AAO90347.1 | No |
| AAO90818.1 | No |
| AAO90822.1 | No |
| AAO90909.1 | No |
| AAO90963.1 | No |
| AAO91013.1 | No |
| AAO91340.1 | No |
| AAO90650.1 | No |

(B)

| **Pos** | **GenBank ID** | **Peptide** | **Gene Name** | **Locus Tag** | **Location** |
| --- | --- | --- | --- | --- | --- |
| 26 | AAO90909.1 | AQMEPRRAL |  | CBU_1411 | CYTOPLASM |
| 4 | AAO91013.1 | KIQPIQQSM |  | CBU_1516 | MEMBRANE(non-PSE) |
| 10 | AAO91340.1 | SAFSASPQF |  | CBU_1849 | CYTOPLASM |

Technical note: The authors acknowledge that the murine MHCI alleles available on the webserver were updated after initial analysis but prior to receiving reviewer feedback. Specifically, alleles H-2-Db, H-2-Dd, H-2-Kb, H-2-Kd, H-2-Kk, and H-2-Ld were consistent between the two runs but H-2-Qa1 and H-2-Qa2 were replaced with H-2-Dq, H-2-Kq, and H-2-Lq. Therefore, the murine MHCI alleles in this reanalysis differ slightly from the remainder of the paper.
